# Autoactive CNGC15 enhances root endosymbiosis in legume and wheat

**DOI:** 10.1038/s41586-024-08424-7

**Published:** 2025-01-15

**Authors:** Nicola M. Cook, Giulia Gobbato, Catherine N. Jacott, Clemence Marchal, Chen Yun Hsieh, Anson Ho Ching Lam, James Simmonds, Pablo del Cerro, Pilar Navarro Gomez, Clemence Rodney, Neftaly Cruz-Mireles, Cristobal Uauy, Wilfried Haerty, David M. Lawson, Myriam Charpentier

**Affiliations:** 1 Cell and Developmental Biology Department, John Innes Centre Norwich Research Park, Norwich, UK; 2 Crop Genetics Department, John Innes Centre Norwich Research Park, Norwich, UK; 3 Biochemistry and Metabolism Department, John Innes Centre Norwich Research Park, Norwich, UK; 4 Earlham Institute, Norwich Research Park, Norwich, UK; 5 Present Address: Microbiology Department, Faculty of Biology, University of Seville, Seville, Spain; 6 Present Address: Department of Plant Biochemistry, Center for Plant Molecular Biology (ZMBP), Eberhard Karls University, Tübingen, Germany; 7 Present Address: University of Pablo de Olavide, Andalusian Center for Developmental Biology/CSIC/Andalusian Government, Seville, Spain; 8 Present Address: Sainsbury Laboratory, University of East Anglia, Norwich Research Park, Norwich, UK

**Keywords:** Rhizobial symbiosis, Arbuscular mycorrhiza, Calcium signalling, Plant breeding

## Abstract

Nutrient acquisition is crucial for sustaining life. Plants develop beneficial intracellular partnerships with arbuscular mycorrhiza (AM) and nitrogen-fixing bacteria to surmount the scarcity of soil nutrients and tap into atmospheric dinitrogen, respectively^1,2^. Initiation of these root endosymbioses requires symbiont-induced oscillations in nuclear calcium (Ca^2+^) concentrations in root cells^3^. How the nuclear-localized ion channels, cyclic nucleotide-gated channel (CNGC) 15 and DOESN’T MAKE INFECTIONS1 (DMI1)^4^ are coordinated to specify symbiotic-induced nuclear Ca^2+^ oscillations remains unknown. Here we discovered an autoactive CNGC15 mutant that generates spontaneous low-frequency Ca^2+^ oscillations. While CNGC15 produces nuclear Ca^2+^ oscillations via a gating mechanism involving its helix 1, DMI1 acts as a pacemaker to specify the frequency of the oscillations. We demonstrate that the specificity of symbiotic-induced nuclear Ca^2+^ oscillations is encoded in its frequency. A high frequency activates endosymbiosis programmes, whereas a low frequency modulates phenylpropanoid pathways. Consequently, the autoactive *cngc15* mutant, which is capable of generating both frequencies, has increased flavonoids that enhance AM, root nodule symbiosis and nutrient acquisition. We transferred this trait to wheat, resulting in field-grown wheat with increased AM colonization and nutrient acquisition. Our findings reveal a new strategy to boost endosymbiosis in the field and reduce inorganic fertilizer use while sustaining plant growth.

## Abstract (web summary)

Nuclear calcium oscillations initiate plant–arbuscular mycorrhiza and nitrogen-fixing bacteria symbioses for nutrient acquisition, with a newly discovered autoactive CNGC15 mutant enhancing these partnerships, potentially improving crop nutrition and reducing inorganic fertilizer dependence.

## Main

Plant cyclic nucleotide-gated channels (CNGCs) have an essential role in the transduction of biotic, abiotic and developmental signals by mediating calcium (Ca^2+^) release, which specifies cellular response^5^. During root endosymbioses, root epidermal cells perceive nitrogen-fixing bacteria and arbuscular mycorrhiza (AM) elicitors through surface-localized receptor-like kinases that induce repetitive Ca^2+^ release into the nucleus^3,6^. Nuclear Ca^2+^ oscillations are essential for activating endosymbiotic gene expression and accommodating endosymbionts^3,7^. In the model legume *Medicago truncatula*, the three paralogues CNGC15a, CNGC15b and CNGC15c, located in a complex with DOESN’T MAKE INFECTIONS1 (DMI1) channel in the nuclear envelope, have been identified as key players in generating Ca^2+^ oscillations^4^. Understanding how CNGC15a, b and c are gated (open and closed) and coordinated with DMI1 in planta to produce symbiosis-specific Ca^2+^ oscillations has been challenging because of their nuclear location. Recently, Ca^2+^-bound calmodulin 2 (holo-CaM2) was shown to close CNGC15a, b and c, providing the negative feedback of the oscillatory behaviour^8^. DMI1 was suggested to activate CNGC15 (ref. ^9^), although the molecular mechanism of CNGC15 opening remains unknown.

CNGCs are formed by four subunits, each containing six transmembrane helices (S1–S6) and a pore loop between S5 and S6 (ref. ^10^). Mammalian CNGCs are gated by intracellular cyclic nucleotide monophosphates (cNMPs) that bind to a cyclic nucleotide-binding domain (CNBD) in the cytoplasmic C terminus and promote opening of the channel^11,12^. Although CNBD is conserved in plant CNGCs^10^, evidence for cNMP regulation in planta is sporadic^13,14^, and alternative mechanisms of opening via CaM have been proposed for a few CNGCs^15^. This suggests that multiple gating mechanisms may coexist within eukaryotic CNGCs to specify their functions. Recently, high-resolution structures of *Caenorhabditis elegans* CNGC in open and closed states revealed that the binding of cNMP to CNBD induces global conformational changes to open the channel, including the movement of S1–S4 transmembrane helices^12^. In legumes, DMI1 interacts with the nucleoplasmic N terminus of CNGC15a, b and c positioned before the S1 helix^4^. Although the role of S1–S4 in gating plant CNGCs has not been explored, this observation suggests that the DMI1-mediated opening of CNGC15a, b and c, might rely on inducing the movement of transmembrane helices.

In this study, we combined Targeting Induced Local Lesions IN Genomes (TILLING) mutant screening and in planta Ca^2+^ imaging to identify a dominant mutation in the S1 helix of CNGC15. This mutation autoactivates the CNGC15 complex in the presence or absence of DMI1, demonstrating that the S1 helix is involved in CNGC15 opening. Autoactive CNGC15 generates a low frequency of Ca^2+^ oscillations that increases plant nutrient acquisition by enhancing colonization by both rhizobia and AM in *M.* *truncatula*. Remarkably, a similar mutation in wheat CNGC15 is sufficient to enhance AM and nutrient acquisition in field conditions. By combining transcriptomic and metabolomic profiling, we further identified the pathway controlled by low-frequency Ca^2+^ oscillation that enhances endosymbioses. Our findings lay the groundwork to provide the activation mechanism of symbiosis-specific Ca^2+^ oscillations and pave the way for future applications to enhance the use of biofertilizers in farming systems.

## CNGC15^GOF^ enhances root nodule symbiosis

**Fig. 1:**
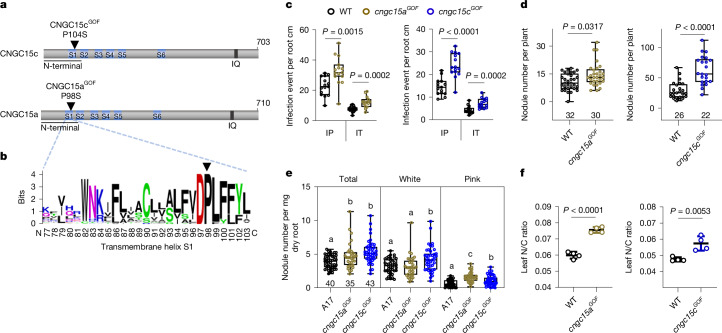
Enhanced root nodule symbiosis in *cngc15a*^*GOF*^ and *cngc15c*^*GOF*^ mutants. **a**, Schematic representation of CNGC15a and CNGC15c, including the calmodulin-binding domain (IQ), the six transmembrane helices S1–S6 and the N-terminal domain. The arrowheads indicate the position of the amino acid substitution P98S in CNGC15a^GOF^ and the corresponding P104S substitution in CNGC15c^GOF^. **b**, Amino acid sequence logo conservation of helix S1 from CNGC encoded within *M.* *truncatula* and *Arabidopsis thaliana*, and CNGC15 encoded within *Glycine max*, *Solanum lycopersicum*, *T. aestivum*, *Oryza sativa*, *Zea mays* and *Arachis hypogaea*. Sequences are indicated in Supplementary Information. **c**, Infection pockets (IPs) and infection threads (ITs) of WT, *cngc15a*^*GOF*^ and *cngc15c*^*GOF*^ 5 dpi with *S.* *meliloti* 2011::*lacZ*. Data represent three biological replicates (*n* = 15). Two-tailed unpaired *t*-test with a previous *F*-test for homoscedasticity. IP (WT versus *cngc15a*^*GOF*^) *P* = 0.0015, IP (WT versus *cngc15c*^*GOF*^) *P* < 0.0001, IT (WT versus *cngc15a*^*GOF*^) *P* = 0.0002 and IT (WT versus *cngc15c*^*GOF*^) *P* = 0.0002. **d**,**e**, Number of nodules formed after 14 (**d**) and 28 (**e**) dpi with *Sm*2011. The numbers under the boxes denote the sample size (*n*). The data represent three biological replicates. **c**–**e**, Box and whisker plots show 25% and 75% percentiles, median, minimum and maximum. **d**, Two-tailed unpaired *t*-test with a previous *F*-test for homoscedasticity. *P* value is indicated. **e**, One-way analysis of variance (ANOVA) with Bonferroni’s multiple comparison test. Different letters indicate statistical significance. Total: (A17 versus *cncg15a*^*GOF*^) *P* = 0.0472, (A17 versus *cncg15c*^*GOF*^) *P* < 0.0001, (*cncg15a*^*GOF*^ versus *cncg15c*^*GOF*^) nonsignificant (NS); white nodules: (A17 versus *cncg15a*^*GOF*^) NS, (A17 versus *cncg15c*^*GOF*^) *P* = 0.0078, (*cncg15a*^*GOF*^ versus *cncg15c*^*GOF*^) *P* = 0.0035; pink nodules: (A17 versus *cncg15a*^*GOF*^) *P* < 0.0001, (A17 versus *cncg15c*^*GOF*^) *P* = 0.0174, (*cncg15a*^*GOF*^ versus *cncg15c*^*GOF*^) *P* = 0.0030. **f**, Nitrogen (N)-to-carbon (C) ratio in leaves of WT, *cngc15a*^*GOF*^ and *cngc15c*^*GOF*^; 28 dpi with *Sm*2011. Scatter plots show mean ± s.d. Data represent four biological replicates. Two-tailed unpaired *t*-test with a previous *F*-test for homoscedasticity. *P* value is indicated. Source Data

**Extended Data Fig. 1:**
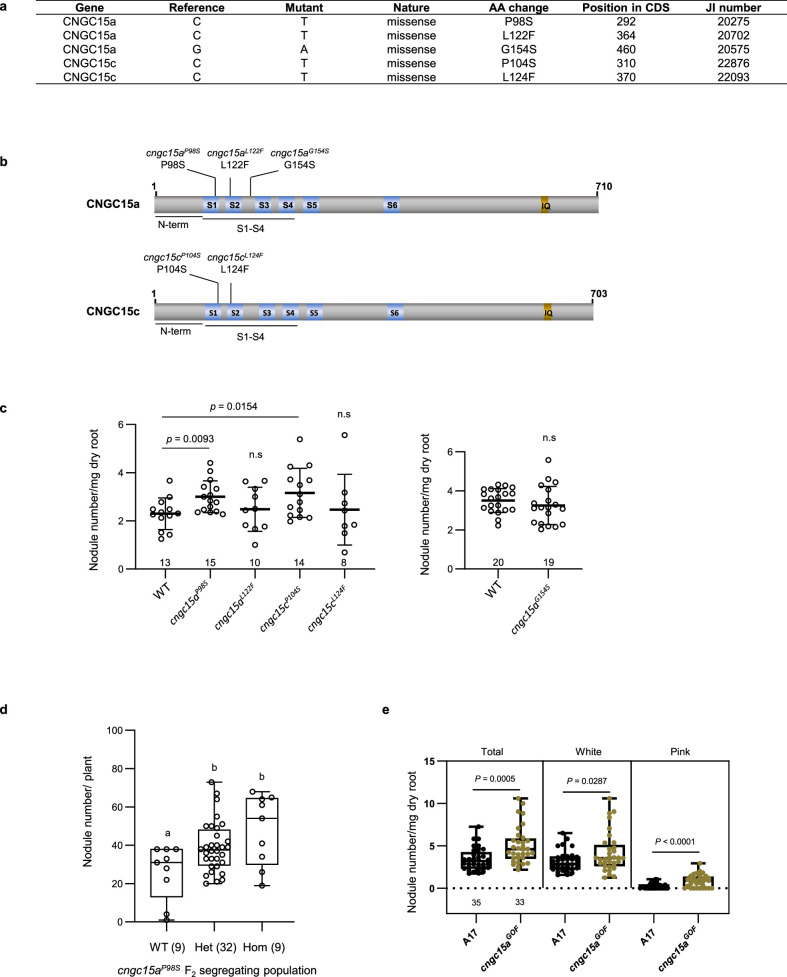
Nodulation phenotyping of *cngc15* TILLING mutants. **a**, TILLING mutant in *S1-S4* sequence of *CNGC15a* and *CNGC15c*. **b**, Schematic representation of CNGC15a and CNGC15c including the calmodulin binding domain (IQ) and the six transmembrane helices S1 to S6. The amino acid substitutions in the *cngc15* mutants analysed in this study are indicated and located within the helices S1 and S2. **c**, Number of nodules observed in *cngc15* mutants 21 dpi with *S. meliloti* 2011. Scatter dot plots show mean ± s.d. Numbers indicate sample size (n), two-tailed unpaired *t*-test with a prior F-test for homoscedasticity. **d**, Number of nodules per plant in F_2_ segregating population of *cngc15a*^*P98S*^ backcrossed with WT. Numbers in brackets indicate number of plants genotyped WT, heterozygous (Het) and Homozygous (Hom). Box and whisker plots show 25% and 75% percentiles, median, minimum, and maximum (One way ANOVA with Tukey’s multiple comparison test, different letters indicate significant difference with (F_2_ WT vs F_2_ Het) *p* = 0.0472, (F_2_ WT vs F_2_ Hom) *p* = 0.0056, (F_2_ Het vs F_2_ Hom) *p* = 0.2395). **e**, Number of nodules formed after 14 dpi with *Sm*2011, including the number of white and pink nodules. Number under boxes denotes sample size (n). The data represent three biological replicates. Box and whisker plots show 25% and 75% percentiles, median, minimum and maximum. Two-tailed unpaired *t*-test with a prior F-test for homoscedasticity. (Total) *p* = 0.0005, (White) *p* = 0.0287, (Pink) *p* < 0.0001. Source Data

To identify missense mutations that could influence CNGC15a, b and c gating activity, we reverse-screened the TILLING mutant collection from *M.* *truncatula*^16^ for mutations in the S1–S4 helices of *CNGC15a* and *CNGC15c*. Among the induced missense mutations retrieved (five in total) (Extended Data Fig. 1a), we identified identical mutations located in the S1 and S2 helices of CNGC15a (P98S and L122F) and CNGC15c (P104S and L124F) (Extended Data Fig. 1b). To investigate whether these mutations could affect root nodule symbiosis, the nodulation phenotype of the homozygous mutant lines was investigated following inoculation with *Sinorhizobium meliloti* 2011 (*Sm*2011). Both *cngc15a*^*P98S*^ and *cngc15c*^*P104S*^, in which the strictly conserved proline is replaced with a serine in the S1 helix (Fig. 1a,b), had significant increases in the number of nodules formed 21 days after inoculation with *Sm*2011 (Extended Data Fig. 1c). We backcrossed *cngc15a*^*P98S*^ to the wild type (WT) and segregated the mutation from the nodulation phenotype. Both homozygous and heterozygous *cngc15a*^*P98S*^ showed an increased number of nodules, demonstrating that the mutation confers a dominant and positive effect and is therefore a dominant gain-of-function (GOF) mutation (Extended Data Fig. 1b). Further analyses of the backcrossed homozygous mutant lines *cngc15a*^*P98S*^ and *cngc15c*^*P104S*^ revealed that the GOF mutations significantly increased the formation of both infection pockets and infection threads 5 days post-inoculation (dpi) and nodule number, including nitrogen-fixing pink nodules, at 14 and 28 dpi with *Sm*2011 (Fig. 1c–e and Extended Data Fig. 1e). Remarkably, the increase in root nodule symbiosis correlated with an increase of more than 20% in the leaf nitrogen (N)-to-carbon (C) ratio (Fig. 1f). Our data collectively indicate that GOF mutations in the S1 helix of either CNGC15a or CNGC15c are sufficient to enhance rhizobial infection, root nodule symbiosis and nutrient acquisition.

## CNGC15^GOF^ autoactivates Ca^2+^ oscillation

**Fig. 2:**
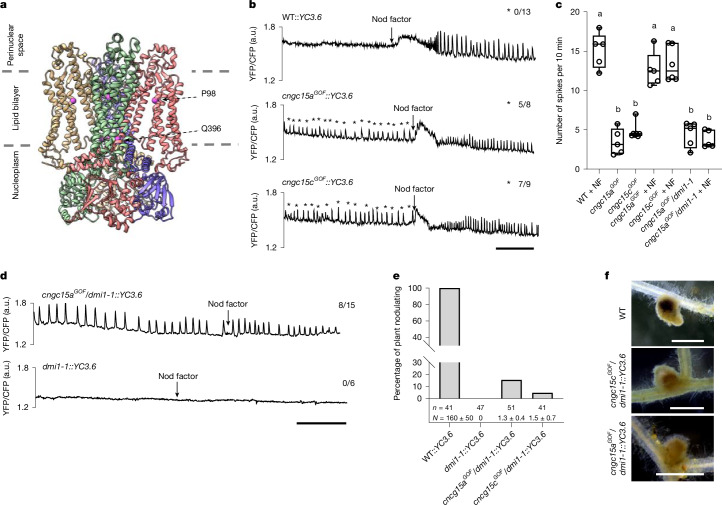
CNGC15^GOF^ autoactivates low-frequency Ca^2+^ oscillations. **a**, Representation of the AlphaFold2 model of CNGC15a, with subunits illustrated in different colours. The grey horizontal dashed lines delineate the expected extent of the lipid bilayer. The positions of residue P98 in S1 and residue Q396 at the channel gate are indicated by the magenta spheres. **b**,**d**, Representative Ca^2+^ oscillations recorded in YC3.6-expressing lines of WT, *cngc15a*^*GOF*^ and *cngc15c*^*GOF*^ (**b**) and *cngc15a*^*GOF*^/*dmi1-1* (**d**) before and after addition of Nod factor (NF) (10^−8^ M). The traces show the ratio of yellow fluorescent protein (YFP)/cyan fluorescent protein (CFP) fluorescence in arbitrary units (a.u.). **b**, Stars indicate spontaneous Ca^2+^ oscillations, and the numbers of plants responding over the total number of plants recorded are indicated on the right. **c**, Analyses of the Ca^2+^ oscillation frequency before and after the NF recorded in **b** and **d**. Box and whisker plots show 25% and 75% percentiles, median, minimum and maximum. One-way ANOVA with Bonferroni’s multiple comparison test. Different letters indicate statistical significance; *P* < 0.0001. **e**, Percentage of plant nodulating after 70 dpi with *Sm*2011/*lacZ*. *n* and *N* indicate the number of plants analysed and the average number of nodules per nodulating plant ± s.d., respectively. (WT::YC3.6) *n* = 41, (*dmi1-1*::YC3.6) *n* = 47, (*cncg15a*^*GOF*^/*dmi1-1*::YC3.6) *n* = 51 and (*cncg15c*^*GOF*^/*dmi1-1*::YC3.6) *n* = 41. **f**, Representative pictures of infected nodules formed in WT and double mutants *cngc15a*^*GOF*^*/dmi1-1*::YC3.6 and *cngc15c*^*GOF*^*/dmi1-1*::YC3.6. Scale bars, 10 min (**b**,**d**), 1 mm (**f**). Source Data

**Extended Data Fig. 2:**
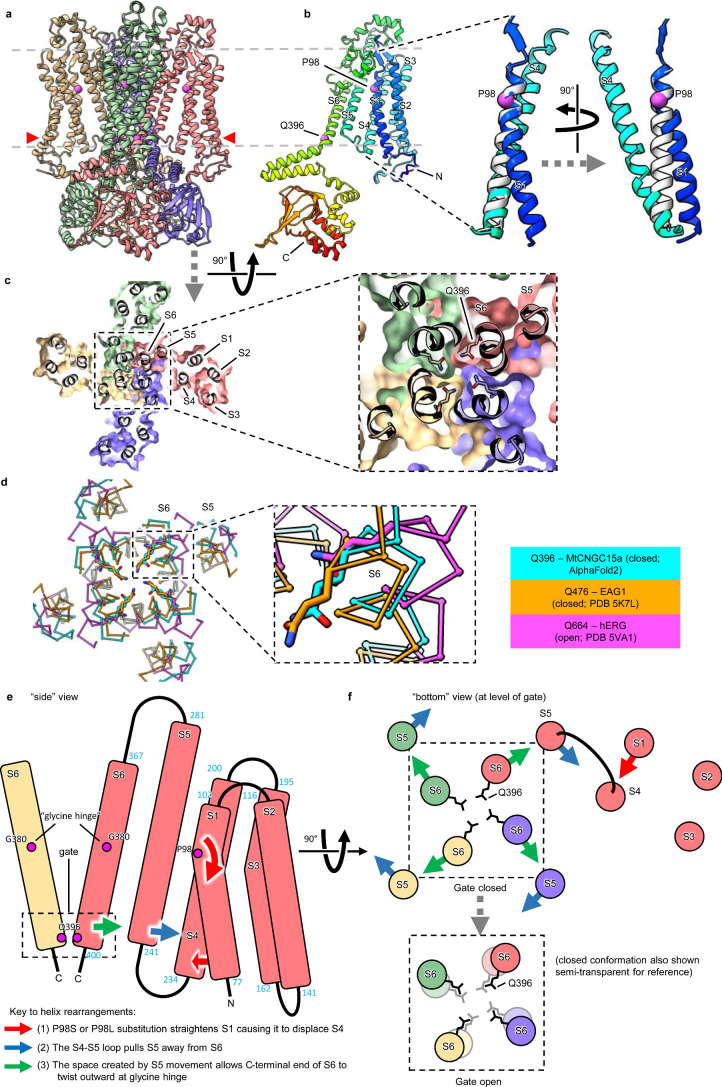
P98S or P98L substitutions are predicted to open CNGC15a gate. **a**, Cartoon representation of the CNGC15a homotetramer predicted using AlphaFold2 with the subunits shown in different colours; the expected extent of the lipid bilayer is delineated by the grey horizontal dashed lines. **b**, Single subunit corresponding to the salmon-coloured chain in (**a**) shown in rainbow colouration from blue at the N-terminus through to red at the C-terminus. The positions of the residues Pro98 and Gln396 are indicated by magenta spheres; the six transmembrane helices are labelled S1-S6. Note the kink in S1 at Pro98. Also shown enlarged are orthogonal views of S1 and S4, illustrating the predicted effect of the P98S (or P98L) substitution that imposes ideal helical geometry on S1 (in grey) whilst keeping it anchored at the upper end, resulting in a clash with the lower end of S4. **c**, A thin slice through a molecular surface of the model, as viewed from below with respect to the view shown in (**a**) and at the level of the channel gating residues Q396 (indicated by the red triangles in (**a**)). The inset close-up clearly shows CNGC15a gating residues Q396. **d**, A superposition of the MtCNGC15a model (cyan carbons) with experimental structures of EAG1 (orange carbons) and hERG (magenta carbons), showing the same view as the (**c**) inset, but with a thicker slab setting; all models are shown as Cα traces with the side-chains of the gating glutamine residues as sticks. The inset close-up emphasises that the conformation of the wild type MtCNGC15a model more closely resembles the closed conformation of EAG1 than the open conformation of hERG. **e**, Schematic representation of the main transmembrane helices of the salmon-coloured subunit in (**a**), with connections shown as simple loops (lengths can be inferred from residue numbering at the ends of each helix in cyan). Helix S6 from the opposing subunit is shown is in pale yellow to highlight the location of the channel. **f**, View from below at the level of the gate, corresponding to that shown in (**c**). For simplicity, the full complement of helices is shown for the right-hand subunit only (salmon). The coloured arrows indicate possible structural changes caused by the P98S/P98L substitution (see key for details) that result in channel opening as illustrated in the lower panel.

**Extended Data Fig. 3:**
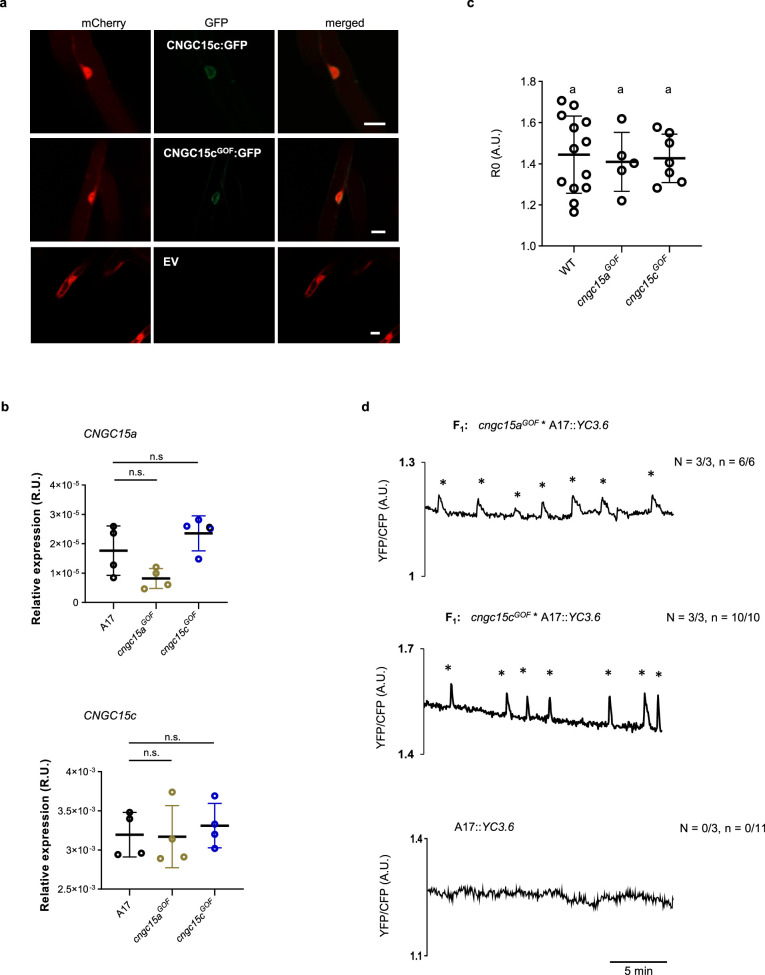
Nuclear-localized CNCG15^GOF^ generates spontaneous nuclear Ca^2+^ oscillation in F_1_ hybrid roots. **a**, CNGC15^GOF^ localizes at the nuclear envelope. Representative images of *M. truncatula* root hairs expressing C-terminal green fluorescent protein (GFP) tagged CNGC15c^GOF^ under the control of nopaline synthase promoter (pNOS) or the empty vector (EV) with the red fluorescent protein (mCherry) used as fluorescent plant transformation marker. The pictures are representative of 4 biological replicates. Scale bars = 10 µm. **b**, Expression analysis of *CNGC15a* and *CNGC15c* by qRT-PCR in the roots of two days old *M. truncatula* wild type (WT), *cngc15a*^*GOF*^ and *cngc15c*^*GOF*^. Expression was normalised to *UBC9* (TC106312). Bars, error bars and circles represent mean, standard deviation, and individual values, respectively, (n = 4). (One-way ANOVA; posthoc Bonferroni). **c**, Scatter dot plot representing the basal CFP/YFP fluorescence ratio (R0) in plants recorded in Fig. 1b. The R0 was computed as the mean of the ratios CFP/YFP obtained during the 23 frames prior Nod factor addition for the wild type (WT), or between spontaneous spike for the mutant *cngc15a*^*GOF*^ and *cngc15c*^*GOF*^. Ratio of YFP/CFP fluorescence is represented in arbitrary units (A.U.). Bar indicates mean and error bar indicates standard deviation (SD). One-way ANOVA; posthoc Tukey, n (WT) = 13, n (*cngc15a*^*GOF*^) = 5, n (*cngc15c*^*GOF*^) = 7. (WT vs. *cncg15a*^*GOF*^*) p* = 0.9132, (WT vs. *cncg15c*^*GOF*^*) p* = 0.9694, (*cncg15a*^*GOF*^ vs. *cncg15c*^*GOF*^*) p* = 0.9832. **d**, Representative recording of spontaneous nuclear Ca^2+^ oscillation in root hair cells of F_1_ hybrid seedlings obtained from cross pollination between *cngc15a*^*GOF*^ (♀) or *cngc15c*^*GOF*^ (♀) and wild type A17::YC3.6 (♂). Ratio of YFP/CFP fluorescence is represented in arbitrary units (A.U.). Numbers on the right corner indicate the number of F_1_ plants (N) and nuclei (n) responding with spontaneous Ca^2+^ oscillation versus the number of plants and nuclei analysed for each genotype. Stars indicate spontaneous Ca^2+^ spike. Source Data

Although comparatively rare in α-helices of water-soluble proteins, proline residues occur more frequently in the transmembrane helices of integral membrane proteins^17^, where they are thought to have key roles in helix packing by introducing local distortions^18^. Consistent with these observations, the AlphaFold2 model of CNGC15a indicates that the conserved proline residue, P98, causes kinking of helix S1 (Fig. 2a and Extended Data Fig. 2a,b). Mutation of the conserved P98 to a serine (S) or leucine (L) is predicted to remove this kink and restore regular helical geometry, which would bring about a rearrangement of neighbouring transmembrane helices (S4–S6) to give a configuration that favours CNGC15 opening at the gate residue Q396 (Extended Data Fig. 2b–f). This suggests that the movement of transmembrane helices is an integral part of the opening mechanism of CNGC15. Supporting this hypothesis, a comparative analysis of the cryo-electron microscopy structures of the CNGC from *C.* *elegans* in closed and open states showed that ligand binding-induced movement of the C-linker causes concerted movement of all transmembrane helices to open the channel^12^. To test whether the GOF mutation, which does not disrupt the localization of CNGC15 at the nuclear envelope (Extended Data Fig. 3a) or its expression level in roots (Extended Data Fig. 3b), was sufficient to open the CNGC15 complex, we monitored Ca^2+^ oscillation in the nuclei of root hair cells of mutant lines expressing the Ca^2+^ reporter Yellow Cameleon version 3.6 (YC3.6). While nuclear Ca^2+^ oscillations were not observed in the WT root hair cells in the absence of rhizobial elicitors (called Nod factors), both mutants exhibited spontaneous Ca^2+^ oscillations, albeit with a frequency significantly lower than that of the Nod factor-induced Ca^2+^ oscillation (Fig. 2b,c). Consistent with the genetic dominance of the GOF mutation, spontaneous Ca^2+^ oscillations, which did not influence the resting Ca^2+^ level (Extended Data Fig. 3c), occurred in the F_1_ hybrids resulting from WT A17::YC3.6 and homozygous *cngc15a*^*GOF*^ or *cngc15c*^*GOF*^ parents (Extended Data Fig. 3d). These results demonstrate that mutation of the conserved proline in the S1 helix of CNGC15a or CNGC15c is sufficient to autoactivate the CNGC15 complex and generate low-frequency nuclear Ca^2+^ oscillations.

## DMI1 is the pacemaker of CNGC15

**Extended Data Fig. 4:**
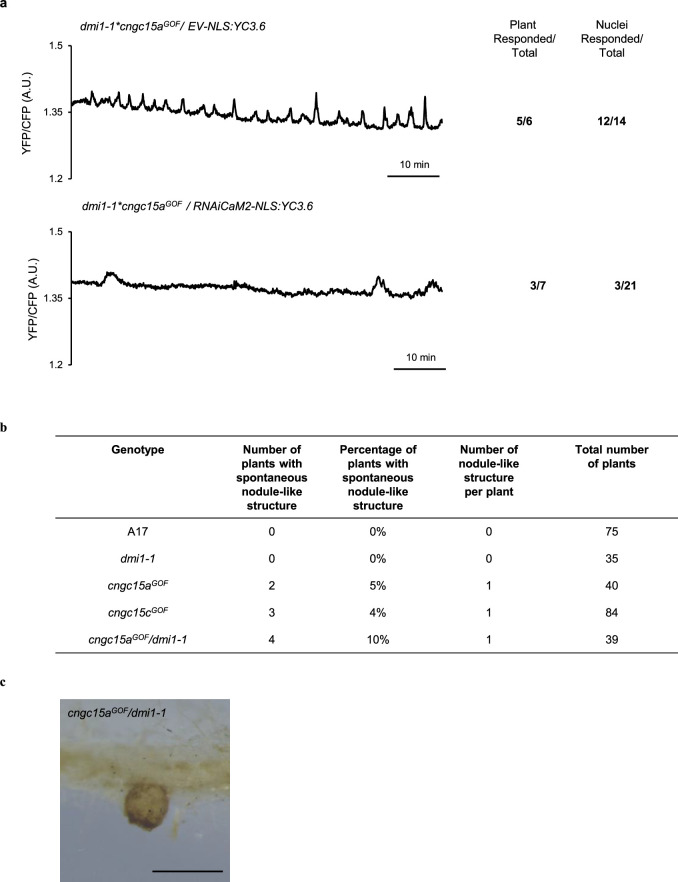
Spontaneous low-frequency nuclear Ca^2+^ oscillation in *cngc15a*^*GOF*^*/dmi1-1* is sustained by the action of CaM2 and is sufficient to restore rare nodule-like structure. **a**, Representative low-frequency Ca^2+^ oscillation in *M. truncatula cngc15a*^*GOF*^/*dmi1-1* double mutant root hair cells co-expressing the nuclear-localized yellow cameleon 3.6 (NLS:YC3.6) alone (EV) or with *RNAiCaM2*. The represented traces show the ratio of YFP/CFP fluorescence in arbitrary units (A.U.). The number of plants and nuclei presenting spontaneous Ca^2+^ oscillations is shown in relation to the total number of plants and nuclei recorded. **b**, Number of plants and nodule-like structures observed on the root system of the wild type *M. truncatula* cv. A17, *dmi1-1*, *cngc15a*^*GOF*^, *cngc15c*^*GOF*^ and the double mutant *cngc15a*^*GOF*^*/dmi1-1* after 98 days of growth. **c**, Representative picture of the nodule-like structure observed. Scale bar indicates 1 cm. Source Data

It was recently suggested that DMI1, which interacts via its C-terminal domain with the N-terminal domain of CNGC15a, b and c^4^, contributes to the activation of the CNGC15 complex in planta^9^. The N-terminal domains of CNGC15a, b and c are located before the S1 helix, where the GOF mutation is positioned (Fig. 1a). We hypothesized that GOF mutations could mimic the movement of transmembrane helices predicted to be induced by DMI1 to activate the CNGC15 complex. In this scenario, CNGC15^GOF^ would be sufficient to generate nuclear Ca^2+^ oscillation in the absence of DMI1. This would further imply that the non-selective cationic permeability of DMI1 is not essential for its symbiotic function in planta. To test these hypotheses, we generated a double homozygous mutant *cngc15a*^*GOF*^/*dmi1-1*::YC3.6, where *dmi1-1* expressing YC3.6 carries a knockout mutation in *DMI1* (ref. ^19^). Spontaneous low-frequency nuclear Ca^2+^ oscillations were recorded in most of the plants tested (Fig. 2d), demonstrating that the GOF mutation can autoactivate CNGC15 in the absence of DMI1. Silencing CaM2 (ref. ^8^), a regulator of CNGC15, impaired the spontaneous nuclear Ca^2+^ oscillations (Extended Data Fig. 4a), demonstrating that in the absence of DMI1, the oscillatory behaviour was sustained by the CaM2-mediated gating of CNGC15. In contrast to *cngc15a*^*GOF*^ and *cngc15c*^*GOF*^, the addition of Nod factor did not restore the WT frequency of Ca^2+^ oscillation in *cngc15a*^*GOF*^/*dmi1-1*::YC3.6 (Fig. 2c,d). In addition, rare infected nodules formed exclusively at the intersections of the lateral and primary roots on *cngc15a*^*GOF*^/*dmi1-1*::YC3.6 and *cngc15c*^*GOF*^/*dmi1-1*::YC3.6 70 dpi with *Sm*2011 (Fig. 2e,f), indicating that infection occurs via crack entry^20^. Consistently, rare spontaneous nodule-like structures were formed in *cngc15c*^*GOF*^/*dmi1-1* after 98 days of growth (Extended Data Fig. 4b,c). Together, these results demonstrate that DMI1 is not only essential for activating CNGC15, but it also acts as a pacemaker for the frequency of nuclear Ca^2+^ oscillations, suggesting that low-frequency Ca^2+^ oscillations cannot restore epidermal infection by rhizobia but are sufficient to induce cortical nodule organogenesis.

**Extended Data Fig. 5:**
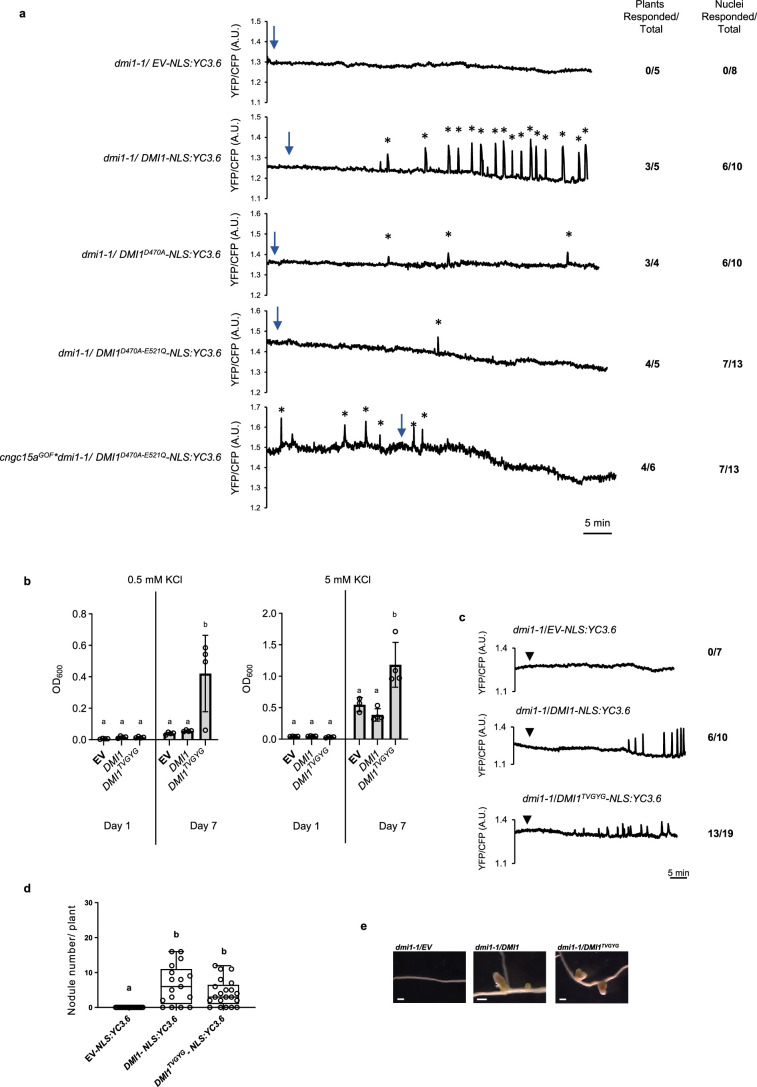
The calcium binding pockets of DMI1 provide the positive feedback to regulate its pacemaker activity and DMI1^TVGYG^ restores nuclear Ca^2+^ oscillation and root nodule symbiosis. **a**, Representative Nod factor-induced Ca^2+^ oscillation in *M. truncatula dmi1-1* mutant root hair cells co-expressing the nuclear-localized yellow cameleon 3.6 (NLS:YC3.6) alone (EV) or with *UBI:DMI1, UBI:DMI1*^*D470A*^*, UBI:DMI1*^*D470A-E571Q*^, and in *M. truncatula cngc15a*^*GOF*^*/dmi1-1* mutant root hair cells co-expressing NLS:YC3.6 with *UBI:DMI1*^*D470A-E571Q*^. The represented traces show the ratio of YFP/CFP fluorescence in arbitrary units (A.U.). The number of plants and nuclei presenting Ca^2+^ oscillations in response to Nod factor are shown in relation to the total number of plants and nuclei recorded. The blue arrow indicates Nod factor application. **b**, Functional complementation assay of the yeast MAB2d mutant strain expressing either the wild type *DMI1* or the *DMI1*^*TVGYG*^ variant, possessing the BK channel filter. The scatter graphs with bar represent the growth of MAB2d strain lacking the potassium transporter Trk1 and Trk2 and transformed with either the empty vector (EV), *pGAL1*:*DMI1* or *pGAL1*:*DMI1*^*TVGYG*^. The growth of the yeast transformants is assessed by the measure of the optical density at 600 nm (OD_600_) after 1 and 7 days of culture in liquid synthetic dropout media lacking tryptophan and supplied with 1% galactose, 1% raffinose and supplemented with the indicated concentration of KCl. Scatter dot plots with bar and error bar indicating mean and standard deviation, respectively. The assays include three biological replicates. Treatments with 0.5 mM KCl were performed with (EV) n = 4, (*DMI1*) n = 4, (*DMI1*^*TVGYG*^) n = 4, and with 5 mM KCl day 1: (EV) n = 4, (*DMI1*) n = 4, (*DMI1*^*TVGYG*^) n = 4, day 7: (EV) n = 3, (*DMI1*) n = 4, (*DMI1*^*TVGYG*^) n = 4, (Two-way ANOVA with Sidak’s multiple comparison). 0.5 mM KCl, 7 days; (EV vs. *DMI1*) non-significant, (EV vs. *DMI1*^*TVGYG*^) *p* = 0.0001, (*DMI1* vs. *DMI1*^*TVGYG*^) *p* = 0.0002. 5 mM KCl, 7 days; (EV vs. *DMI1*) non-significant, (EV vs. *DMI1*^*TVGYG*^) *p* = 0.0002, (*DMI1* vs. *DMI1*^*TVGYG*^) *p* < 0.0001. **c**, Representative Nod factor (10^−8 ^M)-induced Ca^2+^ oscillation in *M. truncatula dmi1-1* mutant root hair cells co-expressing the nuclear localized yellow cameleon 3.6 (NLS:YC3.6) with *UBI:DMI1* or *UBI:DMI1*^*TVGYG*^. The represented traces show the ratio of YFP/CFP fluorescence in arbitrary units (A.U.). The number of nuclei presenting Ca^2+^ oscillations in response to Nod factor (arrowhead) is shown in relation to the total nuclei recorded within three independent transformed plants. **d**, Number of nodules formed on *dmi1-1* mutant roots co-expressing *M. truncatula DMI1* or *DMI1*^*TVGYG*^ driven by the *Lotus japonicus* ubiquitin (*UBI1*) promoter, or the empty vector (*EV)* and the nuclear localized yellow cameleon 3.6 (*NLS*:*YC3.6)*, 28 dpi with *Sm*2011. Box and whisker plots show 25% and 75% percentiles, median, minimum and maximum, of 3 biological replicates. Different letters indicate significant differences (One-way ANOVA; posthoc Bonferroni), n(*EV*) = 19, n(*DMI1*) = 17, n(*DMI1*^*TVGYG*^) = 21. (EV vs *DMI1*) *p* < 0.0001, (EV vs *DMI1*^*TVGYG*^) *p* = 0.0033, (*DMI1* vs *DMI1*^*TVGYG*^) non-significant. **e**, Representative pictures of nodules formed on *dmi1-1* root transcomplemented in (**a**). Scale bar = 1 mm. Source Data

The C-terminal domain of DMI1 is structurally conserved with the C-terminal domain of large-conductance Ca^2+^-activated potassium (BKca) channels in mammals^21^, and it possesses Ca^2+^ binding pockets essential for its function^9^. Mathematical modelling suggests that Ca^2+^ released by CNGC15 provides positive feedback on DMI1 to sustain the Nod factor-induced high Ca^2+^ oscillation frequency^4^. To test whether the Ca^2+^ positive feedback on DMI1 is required for its pacemaker activity, we co-expressed the characterized mutant variants *DMI1*^*D470A*^ or *DMI1*^*D470A-E591Q 9*^, which were mutated in one or two Ca^2+^ binding pockets, respectively, with the nuclear-localized YC3.6, in the roots of the *dmi1-1* mutant. In response to Nod factor, mutation of one Ca^2+^ binding pocket in DMI1^D470A^ drastically impaired the nuclear Ca^2+^ oscillation frequency, whereas mutation of both Ca^2+^ binding pockets in DMI1^D470A-E591Q^ abolished both low-frequency and fast-frequency oscillatory behaviours, but sustained the first Nod factor-induced nuclear Ca^2+^ spike (Extended Data Fig. 5a). This result demonstrates that in response to the Nod factor, activated DMI1 is essential for sustaining a fast frequency of Ca^2+^ oscillation via the Ca^2+^-binding domain within its C-terminal region. Additionally, the presence of activated DMI1^D470A-E591Q^ disrupted the gating of CNGC15^GOF^. This result further suggests that the mobility of the DMI1 C-terminal region in response to Ca^2+^ binding contributes to the pacemaker activity by modulating CNGC15 gating.

To confirm that the non-selective cation permeability of DMI1 is not essential for its function, we swapped the non-selective cationic filter of DMI1 that comprised the motif ‘ADAGN’ with the strictly potassium-selective signature ‘TVGYG’^22–24^, thereby generating DMI1^TVGYG^. Complementation analysis of the yeast mutant MAB2d impaired in active K^+^ uptake^25^ confirmed that DMI1^TVGYG^ permeates potassium (Extended Data Fig. 5b). Using *Agrobacterium rhizogenes*-mediated transformation^26^, we generated stable transformed roots of the *dmi1-1* mutant expressing *DMI1* or *DMI1*^*TVGYG*^. The nuclear-localized YC3.6 was co-expressed to visualize Ca^2+^ oscillation. *DMI1*^*TVGYG*^ restored Nod factor-induced Ca^2+^ oscillations (Extended Data Fig. 5c) and complemented the root nodule symbiosis defect of the *dmi1-1* mutant, as observed 28 days after inoculation with *Sm*2011 (Extended Data Fig. 5d,e). These results demonstrate that substituting a non-selective filter with a potassium-selective filter does not impair the function of DMI1. Collectively, our findings show that mutation of the highly conserved proline residue in the S1 helix of CNGC15a or CNGC15c leads to autoactivation of the CNGC15 complex, resulting in a low frequency of nuclear Ca^2+^ oscillations in the absence of DMI1. However, in the WT, DMI1 is essential for activating the CNGC15 complex and setting the pace of high-frequency nuclear Ca^2+^ oscillations that are required to sustain epidermal infection.

## CNGC15^GOF^ enhances AM colonization

**Fig. 3:**
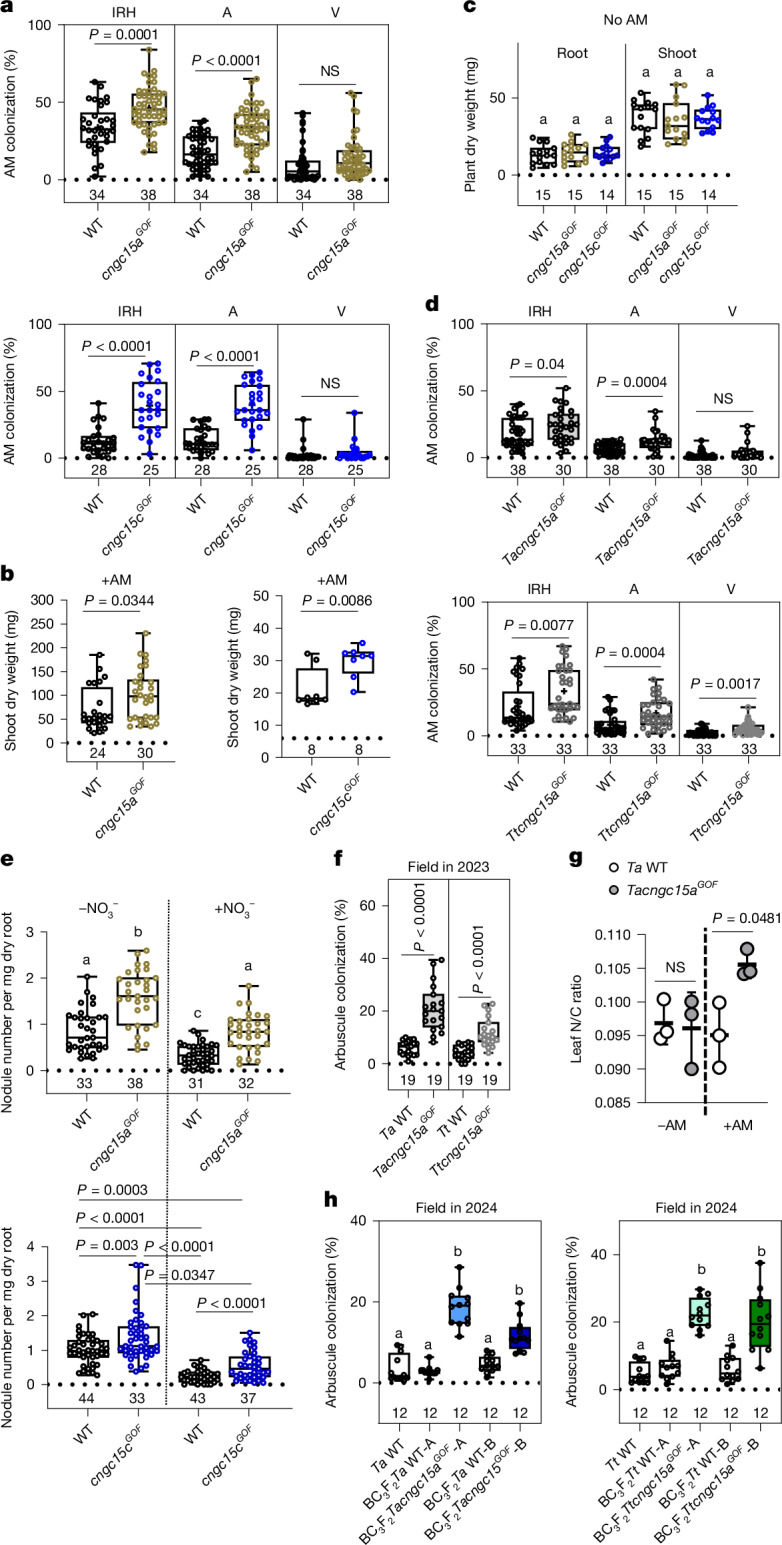
*M.* *truncatula* and wheat *cngc15*^*GOF*^ mutants benefit from enhanced AM symbiosis. **a**, AM colonization, including percentage intraradical hyphae (IRH), arbuscules (A) and vesicles (V) of *M.* *truncatula cngc15a*^*GOF*^ and *cngc15c*^*GOF*^, 5 weeks post-inoculation (wpi) with *R.* *irregularis*. **b**,**c**, Shoot and root dry weight measured 28 days post-growth without *R.* *irregularis* (**c**) and shoot dry weight 28 dpi with *R.* *irregularis* (**b**). **d**, Percentage AM colonization, including IRH, A and V of *T.* *aestivum* cv. *Cadenza* *Tacngc15a*^*GOF*^ and *T. turgidum* cv. *Kronos* *Ttcngc15a*^*GOF*^ under greenhouse-controlled conditions 8 wpi with *R.* *irregularis*. **e**, Number of nodules formed in *M.* *truncatula* WT, *cngc15a*^*GOF*^ and *cngc15c*^*GOF*^ in the presence and absence of 3 mM KNO_3_^−^ 28 dpi with *Sm*2011. **f**, Percentage arbuscule colonization in *T.* *aestivum* cv. *Cadenza* WT (*Ta*WT), *Tacngc15a*^*GOF*^, *T.* *turgidum* cv. *Kronos* WT (*Tt*WT) and *Ttcngc15a*^*GOF*^, 13 wpi with *R.* *irregularis* in the field. **g**, Nitrogen (N)-to-carbon (C) ratio in flag leaf of WT *T.* *aestivum* cv. *Cadenza* and *Tacngc15a*^*GOF*^ from the field experiment (**f**), collected 13 wpi with AM inoculation (+AM) or without (−AM). Values represent three different plots. **h**, Percentage of arbuscule colonization of *Tt*WT, *Ta*WT and two independent BC_3_F_2_ homozygous lines for each *Tacncg15a*^*GOF*^ and *Ttcngc15a*^*GOF*^ (A and B) and their respective BC_3_F_2_ WT, 13 wpi with *R.* *irregularis* in the field. **a**–**f**,**h**, Box and whisker plots show 25% and 75% percentiles, median, minimum and maximum. The number of plants analysed is indicated below the grid line. **a**,**c**,**d**,**f**,**g**, Statistical significance is indicated; two-tailed unpaired *t*-test, previous *F*-test for homoscedasticity. **c**,**e**,**h**, One-way ANOVA with Bonferroni’s multiple comparison test. Different letters indicate statistical significance. **e**, Top panel *P* < 0.0001. **h**, *P* < 0.0001. **a**–**e**, Three biological replicates were performed. Source Data

Symbiotic factor-induced nuclear Ca^2+^ oscillations are essential for both root nodule symbiosis and AM colonization. To assess whether the CNGC15^GOF^-induced low-frequency Ca^2+^ oscillation influences AM colonization, we monitored *Rhizophagus irregularis* colonization of *cngc15a*^*GOF*^ and *cngc15c*^*GOF*^ 5 weeks after inoculation. Similar to root nodule symbiosis, AM colonization was significantly enhanced in both *cngc15a*^*GOF*^ and *cngc15c*^*GOF*^ (Fig. 3a), and this correlated with an increase in shoot dry weight of the mutants (Fig. 3b), whereas no difference in root and shoot dry weights of the mutants in comparison to the WT was observed in the absence of symbionts (Fig. 3c).

**Extended Data Fig. 6:**
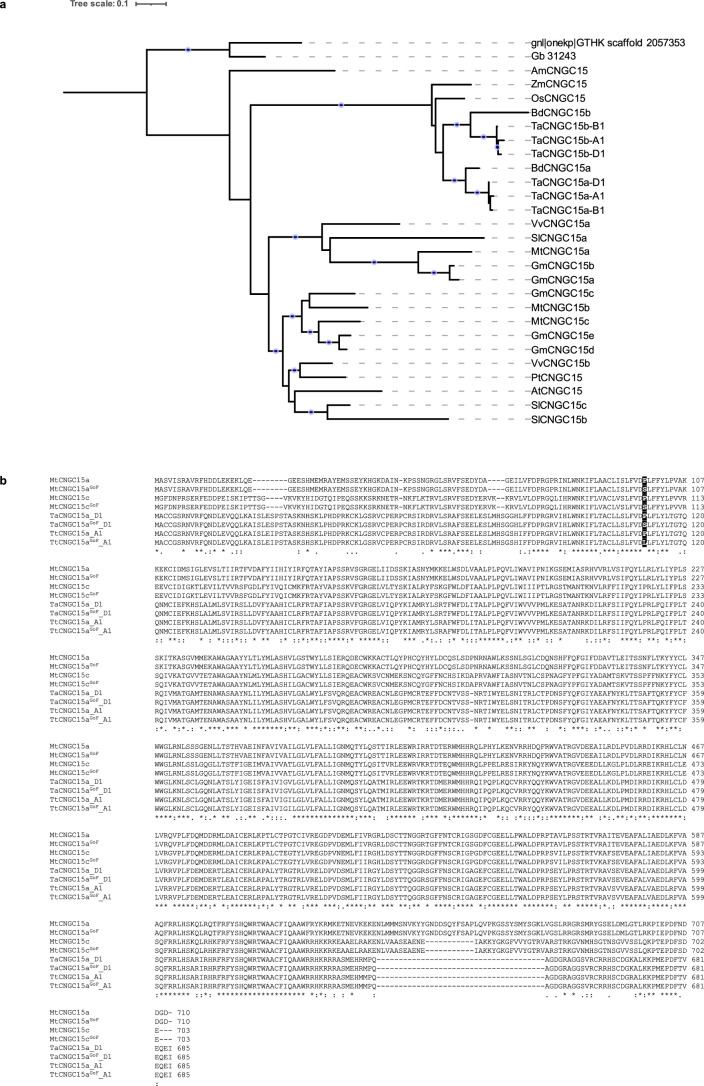
Phylogenetic analyses of the CNGC15 family and amino acid alignment of *Medicago truncatula* and *Triticum sp*. CNGC15a and CNGC15a^GOF^. **a**, Maximum likelihood tree using amino acid sequences under the JTT + G4 substitution model. The sequences used are available in SI Table 3. Ultrafast bootstrap support values over 90 are labelled on the branches as blue dots. The tree is rooted with gymnosperm CNGC clade III sequences. Species abbreviation - At: *Arabidopsis thaliana*; Am: *Amborella trichopoda*; Bd: *Brachypodium distachyon*; Gm: *Glycine max*; Mt: *Medicago truncatula*; Os: *Oryza sativa*; Pt: *Populus trichocarpa*; Ta: *Triticum aestivum*; Sl: *Solanum lycopersicum*; Vv: *Vitis vinifera*; Zm: *Zea mays*. **b**, The mutated sites of MtCNGC15a^GOF^ 104 P > S; MtCNGC15c^GOF^ 98 P > S; TaCNGC15a^GOF^_D1 cv. Cadenza 111 P > S; TtCNGC15a^GOF^_A1 cv. Kronos P111 > L are highlighted.

Considering that the GOF mutation in *CNGC15* is dominant and that AM colonizes most land plants^27^, we next tested whether the enhanced AM benefits conferred by CNGC15^GOF^ could be transferred to agronomically significant species, such as wheat (*Triticum* sp.). The hexaploid bread wheat, *Triticum aestivum*, possesses six *CNGC15* genes (Extended Data Fig. 6a). To identify mutations in the conserved proline in the S1 helix of *Triticum CNGC15a*, we explored the TILLING populations developed in the *T.* *aestivum* cv. *Cadenza* and the tetraploid *Triticum turgidum* cv. *Kronos*^28^ for mutations in *TaCNGC15a* and *TtCNGC15a* homologues. Two lines harbouring a mutation in the conserved proline in the S1 helix of *TaCNGC15a-D1* (D-genome) and *TtCNGC15a-A1* (A-genome) were identified. The *Cadenza* mutant line *Tacngc15a*^*GOF*^ carried a P111S substitution, whereas the *Kronos* mutant line *Ttcngc15a*^*GOF*^ carried a P111L substitution that was predicted to confer the same structural effect as P111S (Extended Data Fig. 6b). Analyses of AM colonization 8 weeks after inoculation with *R.* *irregularis* revealed a significant increase in AM colonization in both the *Tacngc15a*^*GOF*^ and *TtCNGC15a*^*GOF*^ mutant lines under controlled greenhouse conditions (Fig. 3d). These results indicate that the enhanced AM colonization conferred by mutation of the proline residue in helix 1 can be transferred to cereal crops such as polyploid wheat.

## CNGC15^GOF^ sustains symbiosis in fields

Since the mid-1900s, the application of chemical fertilizers has emerged as the main method for boosting crop productivity. However, this practice has inflicted considerable damage on our environment^29,30^; thus, the reduction of inorganic fertilizers within agricultural systems is a considerable challenge^31^. The use of root endosymbionts as a source of natural fertilizer is one of several approaches that can help diminish the rates of application of inorganic fertilizers. However, plants engage more efficiently in symbioses with rhizobia and AM when grown in nutrient-depleted soil. Most arable lands used for intensive farming practices, however, are enriched with residual nitrogen and phosphate, making the use of symbionts as biological fertilizer inefficient^32,33^. To assess whether CNGC15^GOF^ enhances the *M.* *truncatula* root nodule symbiosis in the presence of nitrate, we monitored nodulation of *cngc15a*^*GOF*^ and *cngc15c*^*GOF*^ mutants watered with 3 mM KNO_3_^−^ after inoculation with *Sm*2011. As expected, nodulation in WT plants was significantly inhibited by the addition of nitrate (Fig. 3e). Although nitrate also reduced nodulation of the mutants, the number of nodules was significantly higher than that in the WT, averaging the level of nodulation as WT grown without added nitrate (Fig. 3e). This result indicates that low-frequency Ca^2+^ oscillation induced by CNGC15^GOF^ partially suppressed the inhibition of nodulation by nitrate.

To determine whether AM colonization could be enhanced in wheat mutants grown in field conditions, we evaluated the AM colonization of *Tacngc15a*^*GOF*^ and *TtCNGC15a*^*GOF*^ across five different plots in the field 4 months after inoculation with *R.* *irregularis*. Soil analysis of this field revealed that these plots had an average nitrate content of 244 mg kg^−1^ and a phosphate content of 32 mg l^−1^, which corresponds to levels observed in non-nutrient-depleted soils^32,33^. Remarkably, AM colonization was significantly higher in both *Tacngc15a*^*GOF*^ and *TtCNGC15a*^*GOF*^ than in WT (Fig. 3f) and was sufficiently higher in *Tacngc15a*^*GOF*^ to enhance the N-to-C ratio in the flag leaf (Fig. 3g), the main organ for photosynthesis that contributes 45–58% photosynthetic performance during the grain-filling stage. The increased AM colonization in the wheat GOF mutants in the field was further confirmed the following year, with a significant increase in arbuscule colonization in the BC_3_F_2_ lines carrying the mutant allele compared to the near-isogenic WT controls 4 months after inoculation with *R.* *irregularis* (Fig. 3h). Thus, the field trials consistently revealed that CNGC15^GOF^ has the potential to enhance endosymbioses in cereal crops in field soil containing residual nitrate and phosphate.

## CNGC15^GOF^ enhances flavonoid production

**Fig. 4:**
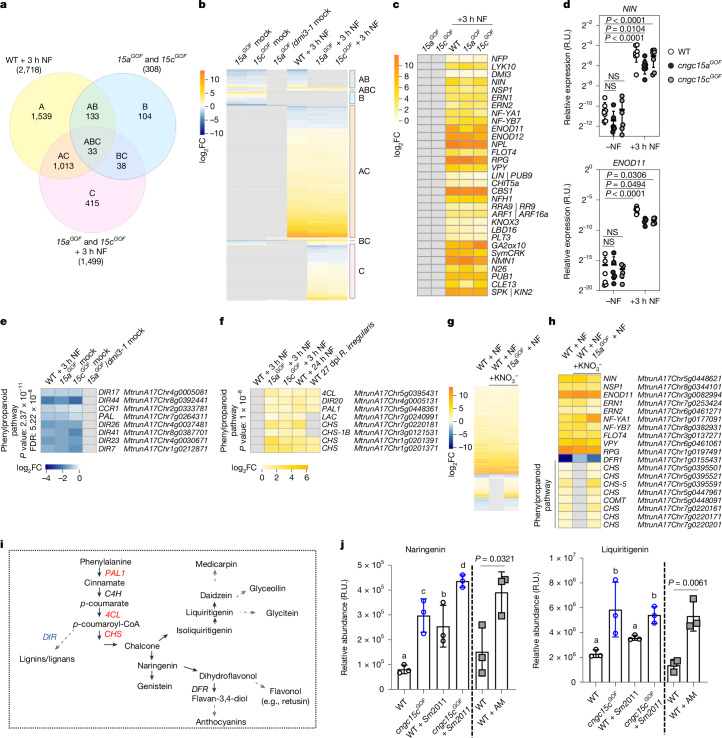
Low-frequency Ca^2+^ oscillations of *cngc15*^*GOF*^ mutants modulate root phenylpropanoid pathways. **a**, Venn diagram showing overlap of differentially expressed genes (DEGs) in *cngc15a*^*GOF*^ (*15a*^*GOF*^) and *cngc15c*^*GOF*^ (*15c*^*GOF*^) versus WT (308 genes), *cngc15a*^*GOF*^ and *cngc15c*^*GOF*^ treated with Nod factor (NF) for 3 h versus mock (1,499 genes) and WT treated with NF for 3 h versus mock (2,718 genes) (*P* value < 0.05). **b**, Heat map showing DEGs in *15a*^*GOF*^ and *15c*^*GOF*^ mock or in response to NF identified in **a** that overlaps with DEGs in WT (clusters AB, ABC and AC) or not (clusters B, BC and C) and in *15a*^*GOF*^*/dmi3-1* mock. **c**, Heat map showing the log_2_ fold change (FC) of endosymbiotic genes, which are not induced in *15a*^*GOF*^ and *15c*^*GOF*^ in the absence of NF. **d**, Quantitative expression analysis of *NIN* and *ENOD11* relative to *UBC9*, with (+) and without (−) 3 h NF; values from three biological replicates. **e**–**h**, Heat map showing the log_2_FC of genes over-represented in either cluster AB–ABC (**e**), cluster C (**f**) or DEGs in response to NF and 3 mM nitrate treatment (**g**), including *CHS* (**h**). **e**,**f**, Fisher’s exact test, two-tailed, false discovery rate (FDR) < 5 × 10^−4^. **g**,**h**, *P* value < 0.05. **i**, Schematic representation of core phenylpropanoid pathways; phenylalanine ammonia lyase (PAL), cinnamic acid 4-hydroxylase (C4H), 4-coumarate:CoA ligase (4CL), chalcone synthase (CHS), dirigent protein (DIR) and dihydroflavonol reductase (DFR). DEG of *cngc15*^*GOF*^ upregulated, red; downregulated, blue. The dark arrow indicates one step; the grey dashed arrow indicates multiple enzymatic steps. **j**, Relative abundance of naringenin and liquiritigenin in three biological replicates of WT and *cngc15c*^*GOF*^ roots after 21 days of growth in the presence or absence of *Sm*2011 (optical density at 600 nm (OD_600_) = 0.01), and in WT roots after 5 weeks of inoculation with *R.* *irregularis* (AM). **d**,**j**, Scatter plots show mean, s.d. One-way ANOVA, Dunnett’s multiple comparison test versus WT. **d**, Different letters indicate statistical difference (**j**). **j**, Two-tailed unpaired *t*-test with a previous *F*-test for homoscedasticity. RNA-seq reads in **b**, **e** and **f** reproduced from ref. ^34^, Springer Nature, under a Creative Commons licence CC BY 4.0, and ref. ^35^, American Association for the Advancement of Science. Source Data

**Extended Data Fig. 7:**
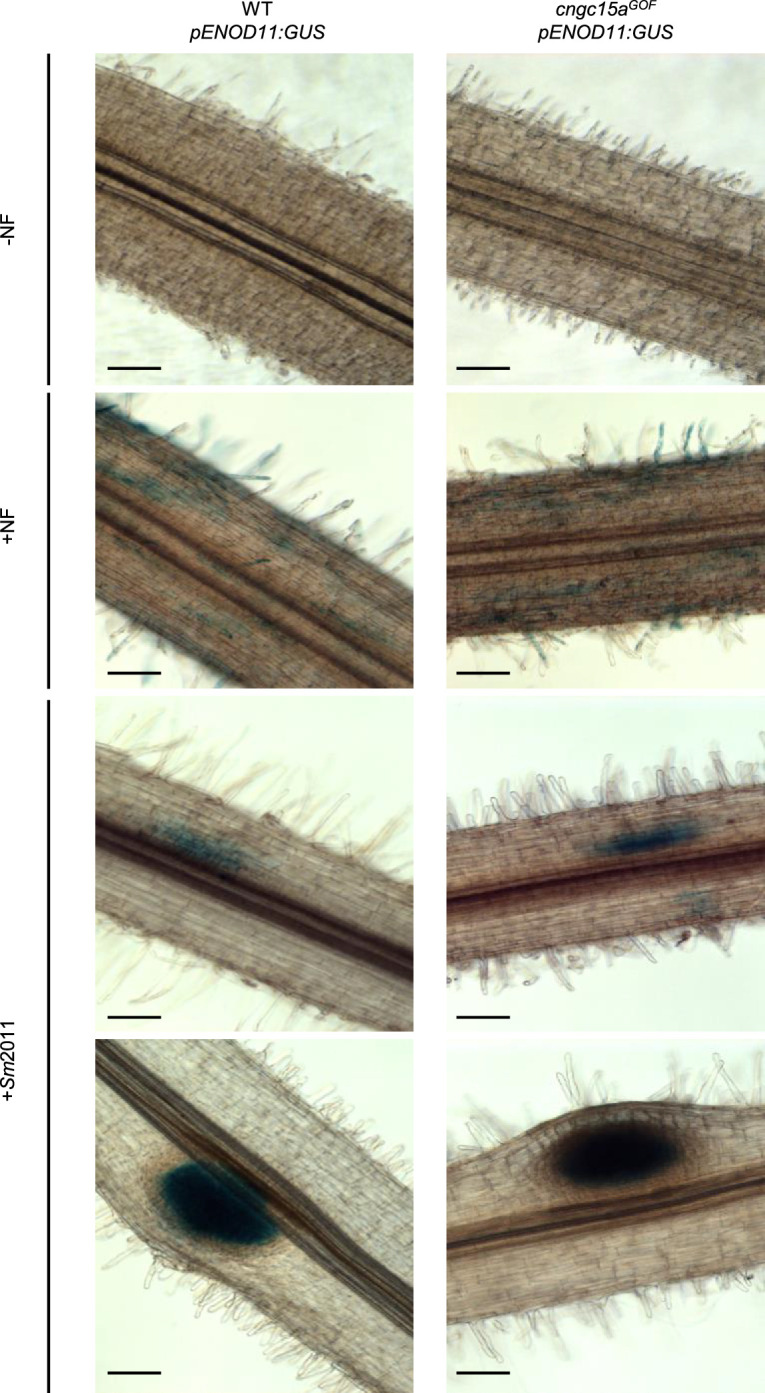
*cngc15a*^*GOF*^ does not activate *ENOD11* expression in the absence of Nod factor. β-glucuronidase (GUS) activity in root induction zones of *M. truncatula* wild type (WT) expressing p*ENOD11:GUS* and *cngc15a*^*GOF*^ expressing p*ENOD11:GUS*, 6 h after incubation with NF (+NF), without NF (-NF) and 6 dpi with *S. meliloti* 2011. Scale bar = 100 µm. Representative pictures of plants from one of three biological replicates, with each replicate consisting of n = 5 plants per treatment.

**Extended Data Fig. 8:**
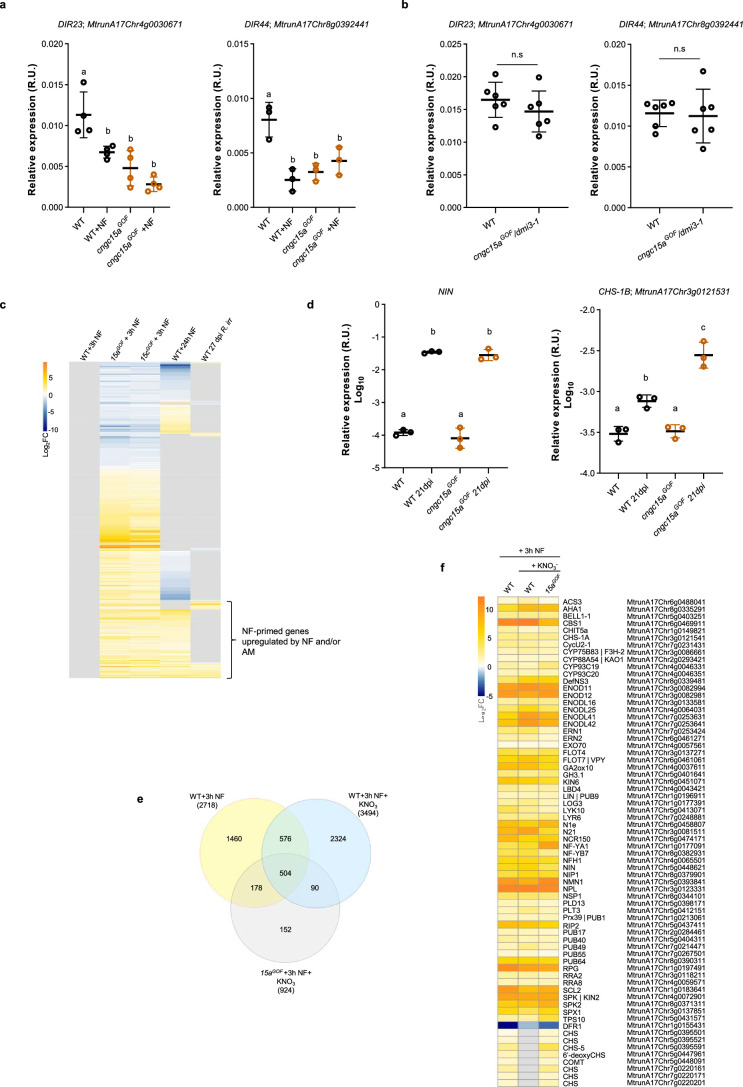
*cngc15*^*GOF*^ primes phenylpropanoid pathways. **a**, **b** Expression analysis of *DIR23* and *DIR44* by qRT-PCR in root of wild type (*WT*),*cngc15a*^*GOF*^ and *cngc15a*^*GOF*^*/dmi3-1* treated or not with 10^−8^ M Nod factor (+NF). Expression was normalised to *UBC9* (TC106312). Bars, error bars and circles represent mean, standard deviation and individual values, respectively, with *DIR23*, n = 4; *DIR44*, n = 3 in (**a**), and *DIR23*, *DIR44*, n = 6 in (**b**). (**a**) Different letters indicate significant differences (One-way ANOVA; posthoc Bonferroni). *DIR23*; *MtrunA17Chr4g0030671* (WT vs. WT + NF) *p* = 0.0273, (WT vs. *cncg15a*^*GOF*^) *p* = 0.002, (WT vs. *cncg15a*^*GOF*^ + NF) *p* = 0.0002. *DIR44*; *MtrunA17Chr8g0392441* (WT vs. WT + NF) *p* = 0.0031, (WT vs. *cncg15a*^*GOF*^) *p* = 0.0075, (WT vs. *cncg15a*^*GOF*^ + NF) *p* = 0.0301. (**b**) Two-tailed unpaired t-test with a prior F-test for homoscedasticity. n.s. indicates non-significant. **c**, Heatmap showing the Log_2_FC of the DEGs in roots of *cngc15a*^*GOF*^ and *cngc15c*^*GOF*^ that belong to cluster C (See Fig. 4a,b) and their expression after 24 h Nod factor (NF) treatment and 27 dpi with *R. irregularis*. (*R.irr*.). Wald test, two-sided, *p-*value < 0.05. **d**, Expression analysis of *NIN* and *CHS-1B* by qRT-PCR in root of wild type (*WT*) and *cngc15a*^*GOF*^ 21 dpi with *Sm2011*. Expression was normalised to *UBC9* (TC106312). Bars, error bars and circles represent mean, standard deviation, and individual values, respectively, (n = 3). Different letters indicate significant differences (One-way ANOVA; posthoc Bonferroni). *NIN*: (WT vs. WT21 dpi) *p* < 0.0001, (WT vs. *cncg15a*^*GOF*^) non-significant (n.s), (WT vs. *cncg15a*^*GOF*^ 21 dpi) *p* < 0.0001, (WT 21 dpi vs. *cncg15a*^*GOF*^) *p* < 0.0001, (WT 21 dpi vs. *cncg15a*^*GOF*^ 21 dpi) n.s., (*cncg15a*^*GOF*^ vs *cncg15a*^*GOF*^ 21 dpi) *p* < 0.0001. *CHS-1B*: (WT vs. WT21 dpi) *p* = 0.0106, (WT vs. *cncg15a*^*GOF*^) n.s, (WT vs. *cncg15a*^*GOF*^ 21 dpi) *p* < 0.0001, (WT 21 dpi vs. *cncg15a*^*GOF*^) *p* = 0.0168, (WT 21 dpi vs. *cncg15a*^*GOF*^ 21 dpi) *p* = 0.0012, (*cncg15a*^*GOF*^ vs *cncg15a*^*GOF*^ 21 dpi) *p* < 0.0001. **e**, Venn diagram showing the overlapping differentially expressed genes (DEGs) identified by RNAseq analysis between WT treated with Nod factor (NF) and WT or *cngc15a*^*GOF*^ treated with NF in the presence of 3 mM KNO_3_., Wald test, two-sided, *p-value* < 0.05. **f**, Heatmap showing the Log_2_FC of the DEGs that belong to endosymbioses and phenylpropanoid pathways induced by 3 h NF treatment in WT, and in WT and *cngc15a*^*GOF*^ incubated with 3 mM KNO_3_^-^. Wald test, two-sided, *p-*value < 0.05. RNA-seq read in **c** reproduced from ref. ^34^, under a Creative Commons licence CC BY 4.0, and ref. ^35^, American Association for the Advancement of Science. Source Data

The main function of nuclear Ca^2+^ oscillation is to activate endosymbiotic gene expression to accommodate endosymbionts. CNGC15^GOF^ induces spontaneous low-frequency Ca^2+^ oscillations that positively impact root nodules and AM symbioses. Thus, we hypothesized that low-frequency Ca^2+^ oscillations could prime the expression of known endosymbiotic genes in *M.* *truncatula*. To test this hypothesis, we conducted RNA sequencing (RNA-seq) of the root infection zone in WT, *cngc15a*^*GOF*^, *cngc15c*^*GOF*^ and *cngc15a*^*GOF*^*/dmi3-1*. In the latter, *cngc15a*^*GOF*^ was backcrossed with *dmi3-1*, a knockout mutant for the Nod factor-induced nuclear Ca^2+^ oscillation decoder, CCaMK^2^. The plants were subjected to three conditions: non-induced with Nod factor (mock) and induced with 10^−8^ M Nod factor for 3 h in the presence or absence of 3 mM nitrate. Analysis of differentially expressed genes in mock *cngc15a*^*GOF*^ and *cngc15c*^*GOF*^ revealed that low-frequency Ca^2+^ oscillation did not induce the expression of genes with known function in endosymbiotic signalling, rhizobial infection, nodule organogenesis, autoregulation of nodulation or nitrogen fixation (Fig. 4a–c). Quantitative reverse transcription polymerase chain reaction (qRT-PCR) analysis validated that the early nodulation genes *NIN* and *ENOD11* were not induced by the *cngc15a*^*GOF*^ and *cngc15c*^*GOF*^ mutations, and that their expression was not further enhanced after Nod factor treatment (Fig. 4d). Analysis of homozygous *cngc15a*^*GOF*^ backcrossed with *pENOD11::GUS* confirmed that no sporadic spontaneous expression of *ENOD11* occurred in *cngc15a*^*GOF*^ seedling roots (Extended Data Fig. 7). Most of the observed changes in mock *cngc15*^*GOF*^ were downregulation of gene expression dependent on CCaMK (DMI3), including an over-representation of dirigent proteins (DIR) acting in phenylpropanoid pathways (Fig. 4e and Extended Data Fig. 8a,b). Additionally, Nod factor treatment of *cngc15*^*GOF*^ identified a set of genes that were upregulated in comparison with WT (Extended Data Fig. 8c). These included genes whose expression is dependent on CCaMK and mimics that of symbiotic-induced genes upregulated at later stages of endosymbiosis, as revealed by comparison with the published transcriptomic profiles of WT roots treated with Nod factor for 24 h (ref. ^34^) or inoculated with *R.* *irregularis* for 27 dpi (ref. ^35^) (Extended Data Fig. 8c). Interestingly, the phenylpropanoid pathway genes were over-represented in this pool of ‘Nod factor-primed’ genes (Fig. 4f,i). These include enzymes from the central phenylpropanoid pathway (phenylalanine ammonia lyase and 4-coumarate:CoA ligase) and chalcone synthase (*CHS*), which correlate with *CHS* being upregulated during later stages of nodulation in *cngc15*^*GOF*^ (Extended Data Fig. 8d). Comparison of the genes induced by Nod factor in WT and *cngc15*^*GOF*^ in the presence of nitrate revealed that several *CHS* genes were differentially regulated and notably insensitive to nitrate addition in *cngc15*^*GOF*^ (Fig. 4g,h and Extended Data Fig. 8e,f). Altogether, the transcriptomic analysis demonstrated that low-frequency Ca^2+^ oscillation consistently modulates reprogramming of the phenylpropanoid pathway genes otherwise regulated by symbionts.

**Extended Data Fig. 9:**
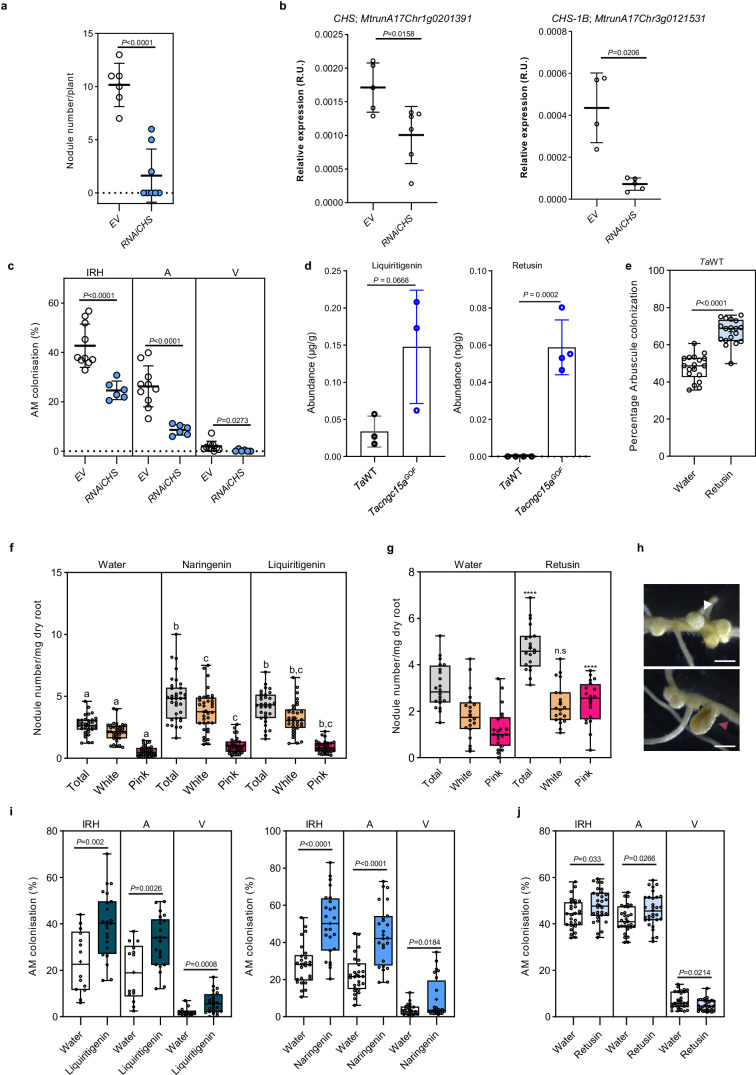
Flavonoids promote endosymbiosis in *M. truncatula* and wheat. **a**, Number of nodules per composite plant expressing the empty vector (*EV*) or the chalcone synthase hairpin construct (*RNAiCHS*) in wild type root systems 21 days post inoculation with *S. meliloti* 2011. **b**, Expression analysis of the chalcone synthase (*CHS*) *MtrunA17Chr1g0201391* and *MtrunA17Chr3g0121531* by qRT-PCR in root analysed in (**a**). Expression was normalised to *UBC9* (TC106312). **c**, Percentage of root length colonization by *Rhizophagus irregularis*, including intraradical hyphae (IRH), arbuscules (A) and vesicles (V) after 5 weeks of inoculation with 1:5 *R. irregularis* inoculum:soil, in roots expressing the empty vector (*EV*) or the chalcone synthase hairpin construct (*RNAiCHS*). **a**-**c**, Scatter dot plot shows the mean ± standard deviation. Two-tailed unpaired t-test with a prior F-test for homoscedasticity. The number of biologically independent samples (n) examined over two biological replicates is as follows: (**a**), EV(n = 6), RNAi*CHS* (n = 8); (**b**), *MtrunA17Chr1g0201391*, EV(n = 5), RNAi*CHS*(n = 6) and *MtrunA17Chr3g0121531*, EV(n = 4), RNAi*CHS*(n = 5); (**c**), EV(n = 10), RNAi*CHS*(n = 6). **d**, Targeted analysis of the abundance of liquiritigenin and retusin in root of wild type *T. aestivum* cv. Cadenza (WT) and *Tacngc15a*^*GOF*^ after 7 weeks of growth in greenhouse conditions. Scatter plots show mean, ± standard deviation. Two-tailed unpaired t-test with a prior F-test for homoscedasticity. The data represent for liquiritigenin three biological replicates and retusin four biological replicates. **e**, External application of 0.1 µM retusin enhances percentage of arbuscule colonization in wild type *T. aestivum* cv. Cadenza (TaWT) after 8 weeks of inoculation with *R. irregularis*. The box and whiskers plots show individual data (n = 18) from three biological replicates, maximum, minimum, median and mean (+). Two-tailed unpaired t-test with a prior F-test for homoscedasticity. **f-h**, Number of nodules per mg of root dry weight of wild type *M. truncatula* 14 days post inoculation with *S. meliloti* 2011 and supplied with water or water containing 0.1 µM naringenin, liquiritigenin or retusin. **f**, **g**, Box and whiskers plots show individual data, boxes bound the interquartile range (25^th^ to the 75^th^ percentile) divided by the median, and whiskers show the maximum, minimum, and mean (+) of the total number of nodules, small white (**h**) or mature pink nodules (**h**). Results represent the average of three biological replicates for naringenin and liquiritigenin (**f**) with n (Water) = 35, n (Naringenin)= 35, n (Liquiritigenin) = 32, and two biological replicates for retusin (**g**) with n (Water) = 20, n (Retusin) = 20. **f**, Different letters indicate significant differences between treatments within the categories of total, small, or mature nodules (One-way ANOVA, Tukey’s multiple comparison). Total (Water vs. Naringenin) *p* < 0.0001, Total (Water vs. Liquiritigenin) *p* < 0.0001, (Naringenin vs. Liquiritigenin) p = 0.1201; White (Water vs. Naringenin) *p* < 0.0001, (Water vs. Liquiritigenin) *p* = 0.0019, (Naringenin vs. Liquiritigenin) *p* = 0.1487; Pink (Water vs. Naringenin) *p* = 0.0004, (Water vs. Liquiritigenin) *p* = 0.0037, (Naringenin vs. Liquiritigenin) *p* = 0.8310. **g**, Two-tailed unpaired t-test with a prior F-test for homoscedasticity. Water vs. Retusin: Total *p* < 0.0001, White *p* = 0.1625, Pink *p* < 0.0001. **h**, Representative pictures of small white and mature pink nodules developed. White and pink arrowheads indicate small and mature nodules, respectively. Scale bar indicates 1 mm. **i**, **j**, Percentage of root length colonization by *Rhizophagus irregularis*, including intraradical hyphae (IRH), arbuscules (A) and vesicles (V) after 5 weeks of inoculation with 1:5 *R. irregularis* inoculum:soil (**i**) or 1:7 *R. irregularis* inoculum:soil (**j**). Box and whiskers plots show individual data, boxes bound the interquartile range (25^th^ to the 75^th^ percentile) divided by the median, and whiskers show the maximum, minimum, and mean (+). Results represent the average of two biological replicates for liquiritigenin (**i**) with n (Water) = 14, n (Liquiritigenin) = 23, three biological replicates for naringenin (**i**) with n (Water) = 26, n (Naringenin) = 24, and three biological replicates for retusin (**j**) with n (Water) = 29, n (Retusin) = 30. Two-tailed unpaired t-test with a prior F-test for homoscedasticity, *p* values indicated. Source Data

**Extended Data Fig. 10:**
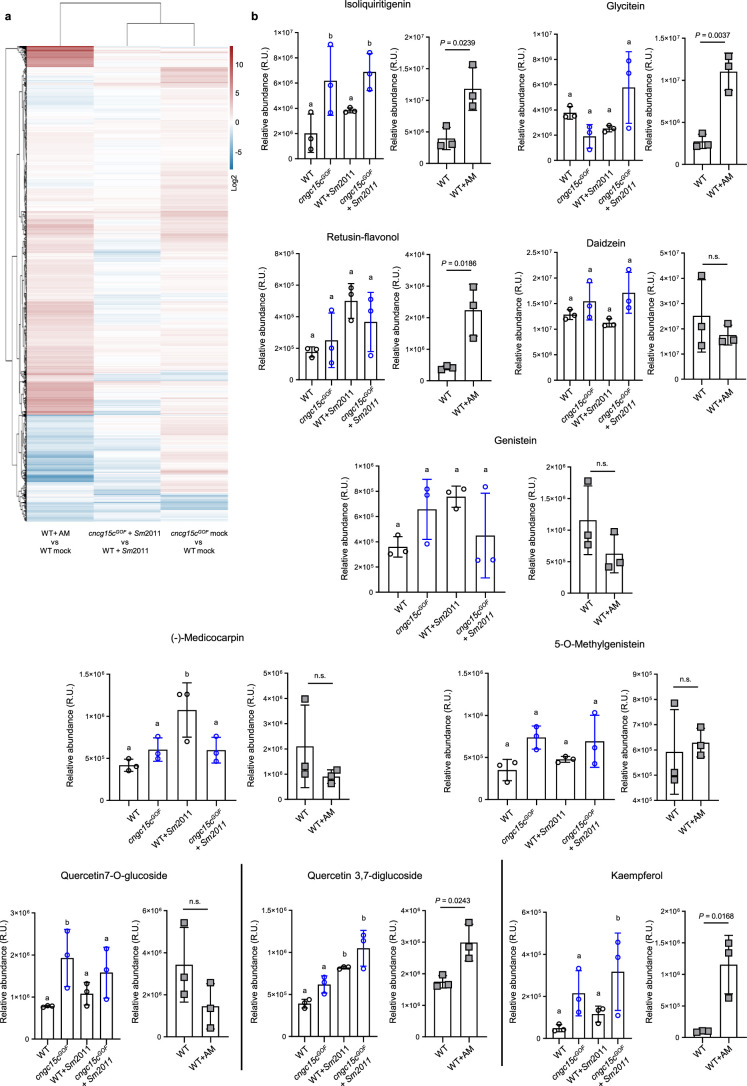
CNGC15^GOF^ enhances flavonoids abundance in *M. truncatula* roots. **a**, Heatmap of the significantly differentially abundant metabolites in roots of wild type (WT) mycorrhized (AM) versus non-mycorrhized (mock), *cngc15c*^*GOF*^ versus WT 21 days post inoculation with *Sm*2011 (+*Sm*2011) or/and *cngc15c*^*GOF*^ versus WT non-inoculated. Limma test, two sided, with a prior Shapiro-Wilk test for homoscedasticity. Post hoc Benjamini and Hochberg. *p*-value < 0.05. **b**, Relative abundance of flavonoids identified across root of wild type (WT) and *cngc15c*^*GOF*^ after 21 days of growth in presence or absence of *Sm*2011 (OD = 0.01). Scatter plots show mean and standard deviation. Results represent three biological replicates. Different letters indicate statistical significance, One-way ANOVA, Dunnett’s multiple comparison test vs. WT. Isoliquiritigenin: (WT vs. *cncg15c*^*GOF*^) *p* = 0.0451, (WT vs. WT + *Sm*2011) *p* = 0.4703, (WT vs. *cncg15c*^*GOF*^ + *Sm*2011) *p* = 0.0219. Glycitein: (WT vs. *cncg15c*^*GOF*^) *p* = 0.3611, (WT vs. WT + *Sm*2011) *p* = 0.6383, (WT vs. *cncg15c*^*GOF*^ + *Sm*2011) *p* = 0.3118. Retusin-flavonol: (WT vs. *cncg15c*^*GOF*^) *p* = 0.8585, (WT vs. WT + *Sm*2011) *p* = 0.0539, (WT vs. *cncg15c*^*GOF*^ + *Sm*2011) *p* = 0.2934. Daidzein: (WT vs. *cncg15c*^*GOF*^) *p* = 0.5571, (WT vs. WT + *Sm*2011) *p* = 0.8202, (WT vs. *cncg15c*^*GOF*^ + *Sm*2011) *p* = 0.2177. Genistein: (WT vs. *cncg15c*^*GOF*^) *p* = 0.2768, (WT vs. WT + *Sm*2011) *p* = 0.1218, (WT vs. *cncg15c*^*GOF*^ + *Sm*2011) *p* = 0.9177. (-)-Medicocarpin: (WT vs. *cncg15c*^*GOF*^) *p* = 0.5413, (WT vs. WT + *Sm*2011) *p* = 0.0085, (WT vs. *cncg15c*^*GOF*^ + *Sm*2011) *p* = 0.5687. 5-O-Methylgenistein: (WT vs. *cncg15c*^*GOF*^) *p* = 0.0747, (WT vs. WT + *Sm*2011) *p* = 0.7335, (WT vs. *cncg15c*^*GOF*^ + *Sm*2011) *p* = 0.1180. Quercetin7-O-glucoside: (WT vs. *cncg15c*^*GOF*^) *p* = 0.0445, (WT vs. WT + *Sm*2011) *p* = 0.7858, (WT vs. *cncg15c*^*GOF*^ + *Sm*2011) *p* = 0.1657. Quercetin 3,7-diglucoside: (WT vs. *cncg15c*^*GOF*^) *p* = 0.1176, (WT vs. WT + *Sm*2011) *p* = 0.0064, (WT vs. *cncg15c*^*GOF*^ + *Sm*2011) *p* = 0.0004. Kaempferol: (WT vs. *cncg15c*^*GOF*^) *p* = 0.2197, (WT vs. WT + *Sm*2011) *p* = 0.7981, (WT vs. *cncg15c*^*GOF*^ + *Sm*2011) *p* = 0.0402. Relative abundance of flavonoids identified across root of WT after 5 weeks of growth in presence or absence of *R. irregularis* (AM), two-tailed unpaired t-test with a prior F-test for homoscedasticity. Isoliquiritigenin (WT vs WT + AM) *p* = 0.0239, Glycitein (WT vs WT + AM) *p* = 0.0037, Retusin-flavonol (WT vs WT + AM) *p* = 0.0186, Quercetin 3,7-diglucoside (WT vs WT + AM) *p* = 0.0243, Kaempferol (WT vs WT + AM) *p* = 0.0168, non-significant (n.s.). Source Data

Phenylpropanoid pathways generate a wide array of secondary metabolites, including plant phenols (for example, lignans) and flavonoids^36^. Flavonoids are well-known to stimulate production of Nod factor by rhizobia^37–39^ as well as AM spore germination and colonization^40^. Notably, silencing of *CHS* was previously shown and confirmed to reduce nodulation in *M.* *truncatula*^41^ (Extended Data Fig. 9a,b). Additionally, expression of the *CHS* hairpin construct in *M.* *truncatula* roots impaired AM colonization (Extended Data Fig. 9c). Most DIR enzymes characterized to date are required to produce plant phenols^42^. These observations, combined with our transcriptomic analysis, suggest that gene expression priming effects modulated by low-frequency Ca^2+^ oscillation could favour the production of flavonoids by the downregulated DIR in mock condition and further enhance flavonoid production by the upregulated *CHS* expression in the presence of endosymbionts. To test whether the production of flavonoids was increased in the *cngc15*^*GOF*^ mutant, metabolomic profiling was performed on 21-day-old roots from *cngc15c*^*GOF*^ and WT inoculated or not inoculated with *Sm*2011 (Extended Data Fig. 10a). Both mycorrhized and non-mycorrhized WT roots were subjected to metabolomic analysis (Extended Data Fig. 10a). Among the metabolites detected, we identified 12 flavonoids (Fig. 4j and Extended Data Fig. 10b). Notably, naringenin, liquiritigenin and isoliquiritigenin were significantly more abundant in *cngc15c*^*GOF*^ roots than in WT, and their production was also significantly enhanced in mycorrhized WT roots (Fig. 4j and Extended Data Fig. 10b). In addition, the production of downstream flavonols, such as retusin (Fig. 4i), was significantly enhanced by AM colonization in *M.* *truncatula* WT roots (Extended Data Fig. 10b) and in the wheat *Tacngc15a*^*GOF*^ root non-inoculated (Extended Data Fig. 9d). External application of naringenin, liquiritigenin or retusin was sufficient to enhance nodulation or AM colonization in *M.* *truncatula* (Extended Data Fig. 9f–j). Similarly, external application of retusin was sufficient to promote AM colonization in WT *T.* *aestivum* cv. *Cadenza* (Extended Data Fig. 9e). Collectively, our transcriptomic and metabolomic data revealed that low-frequency Ca^2+^ oscillations induced by CNGC15^GOF^ modulate phenylpropanoid pathways to enhance the production of core flavonoids and benefit endosymbiotic interactions.

## Discussion

The nuclear-localized CNGC15a, b and c subunits in complex with DMI1 generate symbiotic-specific nuclear Ca^2+^ oscillations that switch on cellular reprogramming to accommodate AM and nitrogen-fixing bacteria. How these two ion channels coordinate the generation of symbiotic-specific nuclear Ca^2+^ oscillations is a long-standing question that has not yet been resolved. In this study, the TILLING mutant screening of *CNGC15a* and *CNGC15c* revealed the importance of the S1 helix in opening CNGC15a, b and c. In CNGC15^GOF^, mutation of the highly conserved proline residue in S1 was predicted to abolish the helix bend, causing a displacement of helices S4, S5 and S6, thereby generating an opening of the pore at the channel-gating residue Q396. This opening of CNGC15^GOF^ is sufficient to generate spontaneous nuclear Ca^2+^ oscillations that are propagated via the binding turnover of holo-CaM2 (ref. ^8^), in the absence of DMI1. However, these spontaneous Ca^2+^ oscillations have a significantly lower frequency than Nod factor-induced Ca^2+^ oscillations and are insufficient to induce the expression of symbiotic genes essential for the accommodation of the symbionts in the epidermis. This demonstrates that the specificity of symbiotic nuclear Ca^2+^ oscillations is encoded in their frequency. Notably, Nod factors induce higher-frequency Ca^2+^ oscillations in *cngc15*^*GOF*^ exclusively in the presence of DMI1, demonstrating that, in addition to unlocking CNGC15 in WT, DMI1 plays the role of a pacemaker via the concomitant action of its C-terminal domain, which binds Ca^2+^ (ref. ^9^) and interacts with the N-terminal domain of CNGC15a, b and c positioned before helix S1 (ref. ^4^).

We discovered that low-frequency Ca^2+^ oscillations modulate the gene expression of the phenylpropanoid pathways correlated with increased flavonoid production in *cngc15*^*GOF*^ roots and an enhancement of root nodule symbiosis and AM colonization. Perception of flavonoids by rhizobia is required to induce the production of Nod factor^39^ and stimulate the bacterial growth rate^43^. Repression of flavonoid production reduces nodulation^44^ and arbuscular mycorrhization. Conversely, the external application of flavonoids increases nodulation in *M.* *truncatula* and AM colonization in *M.* *truncatula* and wheat. Thus, CNGC15^GOF^ can benefit plants by modulating flavonoids and promoting endosymbiotic interactions, which translates to increased nutrient acquisition. *CNGC15* and flavonoid pathways are not restricted to legumes. Remarkably, wheat *cngc15*^*GOF*^ mutants showed enhanced AM colonization in the field and increased nutrient acquisition, even in the presence of inorganic fertilizer. As a result, our discovery holds promise for enhancing AM symbiosis with crops in cultivated soil, thereby reducing the application of chemical fertilizers and associated pollution.

## Methods

### Plant material

*Medicago truncatula* cv. *Jemalong* A17 and A17::YC3.6, *T.* *turgidum* cv. *Kronos* and *T.* *aestivum* cv. *Cadenza* were used as WT. *M.* *truncatula* TILLING alleles were identified from A17 mutant collections (https://www.jic.ac.uk/research-impact/technology-research-platforms/molecular-genetics/medicago-truncatula/)^16^. The population was screened by direct sequencing of target amplicons on a capillary ABI3730 sequencer (Applied Biosystems), as described^16^. The primers used to amplify the target are listed in Supplementary Table 1. *T.* *aestivum* cv. *Cadenza* and *T.* *turgidum* cv. *Kronos* TILLING alleles were identified from the sequenced collections^28^ via Ensembl Plants (www.plants.ensembl.org). The third generation of backcrossed *T.* *aestivum* cv. *Cadenza* mutant and *T.* *turgidum* cv. *Kronos* mutant was generated by manual pollination with their respective WTs. After the third backcross, BC_3_F_1_ plants were self-pollinated, and near-isogenic lines (NILs) homozygous for the GOF mutation and without the mutation (that is, WT) were selected at the BC_3_F_2_ stage. Populations were genotyped as described below in ‘Genotyping of segregating F_2_ population’. F_2_ homozygous and WT *M.* *truncatula cngc15a*^*P98S*^ was backcrossed to WT, and the segregation of the mutations with the phenotype was analysed in F_2_ plants. The *M.* *truncatula cngc15*^*GOF*^ mutant lines were backcrossed to A17::YC3.6, *dmi1-1* (C71)::YC3.6 (ref. ^19^), A17::*ENOD11:GUS*^45^ and *dmi3-1* (ref. ^46^). Transformed roots expressing *DMI1*^*D470A*^*-NLS*:YC3.6, *DMI1*^*D470A-E521Q*^*-NLS*:YC3.6 *DMI1-NLS*:YC3.6 *DMI1*^*TVGYG*^*-NLS*:YC3.6, *RNAiCaM2-NLS*:YC3.6 or *RNAiCHS* were generated via *A.* *rhizogenes*-mediated gene transfer, as described previously^47^ using the *A.* *rhizogenes* strain AR1193.

### Seed sterilization and germination

*M. truncatula* seeds were scarified using sandpaper, treated with 10% sodium hypochlorite for 4 min, washed in sterile dH_2_O and imbibed in sterile dH_2_O for 5 h. The seeds were stratified onto 2% agar for 6 days in darkness at 4 °C and germinated overnight in darkness at 23 °C. For phenotyping experiments, the germinated seedlings were grown on modified Fahraeus medium^48^ in controlled environment for 7 days (23 °C, 16-h photoperiod and 300 µmol m^−2^ s^−1^) before transfer into soil.

*Triticum* sp. seeds were sterilized with 2% sodium hypochlorite for 4 min then washed five times in sterile dH_2_O. The sterilized seeds were stratified for 7 days at 4 °C and germinated for 2 days at 23 °C in darkness. The germinated seedlings were grown on modified Fahraeus medium in a controlled environment for 5 days (23 °C, 16-h photoperiod and 300 µmol m^−^^2^ s^−1^) before transfer into soil.

### Genotyping of segregating F_2_ population

Genomic DNA samples were extracted using DNeasy 96 Plant Kit (Qiagen) according to the manufacturer’s instructions. Single-nucleotide polymorphism of *M.* *truncatula CNGC15*^*GOF*^, *dmi1-1*, *dmi3-1* and *Triticum* sp. *CNGC15*^*GOF*^ was detected via Kompetitive allele-specific PCR^49^ using allele-specific forward primers conjugated with hexachloro-fluorescein fluorescent dye or 6-carboxyfluorescein fluorescent dye, and common reverse primer. The primers used were synthetized by Sigma-Aldrich and are listed in Supplementary Table 1. Reactions were set up in 384-well optically clear plates (4titude Ltd), including 25 ng of genomic DNA, 12 µM of allele-specific primers, 30 µM common reverse primer and 2× PACE master mix (3CR Bioscience). Amplifications were performed in Mastercycler pro 384 (Eppendorf) with the following thermal cycle: 95 °C for 15 min, 10× (95 °C for 20 s; touchdown, 65 °C, −1 °C per cycle and 25 s), 50× (95 °C for 10 s; 57 °C for 60 s). Fluorescent signal data were detected using the PHERAstar plate reader (BMG Labtech). The genotype of homozygous mutants was further confirmed via Sanger sequencing (Genewiz). Genotyping of the *T.* *turgidum* cv. *Kronos* population BC_3_F_2_ was performed using primers P69 and P70, listed in Supplementary Table 1.

Genotyping of *ENOD11:GUS* was performed via PCR using *GUS* specific primers listed in Supplementary Table 1.

### Protein sequence alignment

Amino acid sequences were obtained from the Mt4.0v1 and the International Wheat Genome Sequencing Consortium databases and are listed in Supplementary Table 2. Amino acid sequences were aligned using MUSCLE v.3.8.425 (ref. ^50^). Alignment of the S1 transmembrane domain of CNGC15 is listed in Supplementary Fig. 1.

### Rhizobia infection and nodulation assays

Analysis of infection was performed using the *S.* *meliloti* strain *Sm*2011 (optical density at 600 nm (OD_600_) = 0.01)^51^ transformed with a constitutive hemA-β-galactosidase (*LacZ*) reporter gene fusion (pXLGD4) (ref. ^52^) for visualization. One-day-old seedlings grown on sterile Whatman filter paper and buffered nodulation agar medium (BNM)^53^ containing 0.1 µM l-α-(2-aminoethoxyvinyl) glycine were inoculated and grown, as described previously^4^. For LacZ staining, roots were fixed in 100 mM sodium phosphate (pH 7.0), 10 mM KCl, 1 mM MgCl_2_ and 2.5% glutaraldehyde and stained for β-galactosidase activity overnight in 0.1 M sodium phosphate (pH 7.2), 5 mM K_3_Fe(CN)_6_, 5 mM K_4_Fe(CN)_6_ and 0.02 M 5-bromo-4-chloro-3-indolyl-β-d-galactopyranoside yielding a blue precipitate (Fig. 1c) or 5-bromo-6-chloro-3-indolyl β-d-galactopyranoside yielding a magenta precipitate (Fig. 2f) at 30 °C. Infection threads and infection pockets were scored using a light microscope (Zeiss Axiophot).

For nodulation assays, 1-week-old plants were grown in terragreen/sand (Oil-Dri Ltd) to a ratio (1:1) and inoculated with *Sm*2011 (OD_600_ = 0.01). Plants were grown in a controlled-environment room at 22 °C (80% humidity, 16-h photoperiod and 300 µmol m^−2^ s^−1^). Nodules were scored as indicated. For nitrate experiments, plants were watered with BNM with or without 3 mM KNO_3_.

### Mycorrhization assays

For *M.* *truncatula* mycorrhization experiments, 7-day-old seedlings were transferred in 90% 1:1 sand:terragreen (Oil-Dri Ltd) and 10% mycorrhizal inoculum (homogenized soil substrate containing *Allium schoenoprasum* roots colonized by *R.* *irregularis* DAOM197198). Plants were grown in a controlled-environment room at 22 °C (80% humidity, 16-h photoperiod and 300 µmol m^−2^ s^−1^).

For *Triticum* sp. mycorrhization experiments, 5-day-old seedlings were transferred in 80% 1:1 sand/terragreen mix and 20% mycorrhizal inoculum (as above). Wheat plants were grown in a controlled-environment room at 22 °C (35% daytime humidity and 50% night-time humidity, 16-h photoperiod and 500 μmol m^−2^ s^−1^).

Mycorrhizal fungal structures were visualized after acidic ink staining performed as follows: *M.* *truncatula* roots were cleared in 10% KOH for 5 min at 96 °C, rinsed three times in dH_2_O and stained in acidic ink (5% black ink; Waterman) and 5% acetic acid) for 3 min at 96 °C. *Triticum* sp. roots were cleared in 10% KOH for 25 min at 96 °C, rinsed three times in dH_2_O and stained in acidic ink for 10 min at 96 °C. Roots were destained in 70% chloral hydrate for 10 min and stored in dH_2_O. Mycorrhizal colonization were visualized using an M80 microscope (Leica) and quantified using the grid intersect method^54^.

### Spontaneous nodulation assay

One-week-old *M.* *truncatula* seedlings were planted into 1:1 terragreen:sand (Oil-Dri Ltd) in phytaboxes under sterile conditions. The plants were grown in a controlled-environment room at 22 °C (80% humidity, 16-h photoperiod and 300 µmol m^−2^ s^−1^). Spontaneous nodules were scored after 98 days.

### Shoot and root dry weight

The roots and shoots were separated and dried for 7 days at 28 °C before measuring dry weights with an analytical scale.

### CHN analysis

Leaf tissues were dried for 7 days at 28 °C. Dried leaf material was ground in liquid nitrogen, and CHN elemental microanalysis was performed at Butterworth Laboratories. For *M.* *truncatula*, total dried leaves from five to seven plants were ground per biological replicate. For *Triticum* sp., the dried flag leaf from five to seven plants were ground per plot replicate.

#### Alphafold2 and structural homology modelling

The structure of *M.* *truncatula* CNGC15a homotetramer was predicted with AlphaFold2 multimer, as implemented through ColabFold (v.1.5.2)^55^ (https://colab.research.google.com/github/sokrypton/ColabFold/blob/main/AlphaFold2.ipynb). Four copies of the 710-residue CNGC15a sequence (UniProt accession G7IBJ4) were uploaded to the server, and each of the five independent models was subjected to 20 recycles to improve the accuracy of the predictions. With the exception of regions at the termini of each chain and a few short surface loops, the remainder of the sequence was well predicted according to the quality metrics (Supplementary Fig. 2). After removal of the termini, specifically residues 1–56 and 607–710 in each chain, the remaining core structures gave high overall predicted local distance difference test (pLDDT) scores in the range of 79.7–86.9. Superpositions of the CNGC15a AlphaFold2 models and related structures, as well as prediction of the structural consequence of P98S or P98L, were made using the Secondary Structure Matching algorithm within COOT v.1 (ref. ^56^). In pairwise comparisons, the five MtCNGC15a AlphaFold2 models (truncated as above) gave root-mean-square deviations in the range of 1.099–2.200 Å for the tetramers. The larger values being mostly the result of small changes in the relative tilts of the domains and subunits with respect to one another rather than localized differences. By contrast, superposing the regions encompassing only the transmembrane portion (specifically residues 57–400) of a single subunit from each model gave values in the range of 0.558–0.808 Å. All structural figures were prepared using ChimeraX v.1.5 (ref. ^57^).

### Molecular cloning

The *NLS*:YC3.6, *NLS*:YC3.6*-DMI1*, *NLS*:YC3.6*-DMI1*^*TVGYG*^, *NLS*:YC3.6-*DMI1*^*D470A*^, *NLS*:YC3.6*-DMI1*^*D470A-E521Q*^ and *CNGC15c*^*GOF*^-*mCherry* constructs were generated via Golden Gate cloning according to a previous study^58^. Assembled level 1 modules were cloned in a level 2 binary vector backbone, as presented in Supplementary Fig. 3. Level 0 modules were synthetized by Life Technologies (Thermo Fisher Scientific) except for *DMI1*^*TVGYG*^, *DMI1*^*D470A*^, *DMI1*^*D470A-E521Q*^ and *CNGC15c*^*GOF*^, which were generated via site directed mutagenesis using the primers P32/P33 for *DMI1*^*TVGYG*^, P56/P57 for *DMI1*^*D470A*^, P56–P59 for *DMI1*^*D470A-E521Q*^ and P34–P37 for *CNGC15c*^*GOF*^ followed by Golden Gate cloning to generate the level 0 module. The primer sequences are indicated in Supplementary Table 1. The non-selective selectivity filter of DMI1 was mutated to the potassium selectivity filter identical to KcsA^22^, MthK^24^ and BK channels^23^. The amino acid positions 292–296 of MtDMI1 was mutated from ‘ADAGN’ to ‘TVGYG’.

The previously generated silencing construct *RNAiCaM2* in pK7GWIWG2D(II)^8^ was modified as follows to assess the nuclear calcium oscillation. The *pAtUBI:DsRed* expression cassette was substituted by *pAtUBI:NLS*:YC3.6 via restriction digestion using AatII and ApaI (New England Biolabs) and ligated with T4 ligase (New England Biolabs) to obtain the pK7GWIWG2D(II)YC3.6 empty vector and pK7GWIWG2D(II)YC3.6::*RNAiCAM2*.

The *RNAiCHS* construct was generated using BP/LR Gateway cloning (Invitrogen). The sequence published in a previous study^41^ was amplified from *M.* *truncatula* cv. *Jemalong* A17 roots cDNA using the primers P60/P61 and cloned into the donor vector pDONR207 and subsequently into the destination vector pK7GWIWG2D(II)R following the manufacturer’s instructions (Invitrogen).

### Analyses of Ca^2+^ oscillation

Ca^2+^ oscillation measurements were performed using a Nikon ECLIPSE FN1 equipped with an emission image splitter (OptoSplit II; Cairn Research) and an electron multiplying cooled charge coupled (Rolera Thunder EMCDD) camera (QImaging). enhanced cyan fluorescent protein (ECFP) was excited using light-emitting diode (OptoLED; Cairn Research) at 436 ± 20 nm and emitted fluorescence detected at 535 ± 30 nm (cpVenus) and 480 ± 40 nm (ECFP). Images were collected in 3-s intervals for 1 h and 30 min using MetaFluor software. Two-day-old seedlings or 2-week-old transformed roots were placed in a chamber made on a 48 × 64-mm coverglass (Solmedia) using high-vacuum grease (Dow Corning GmbH). The chamber was filled with 1 ml BNM. Only the root was covered with a coverslip to leave space for *S.* *meliloti* Nod factor application at a final concentration of 10^−8^ M. Nod factor was produced as described previously^59^. Ca^2+^ imaging was performed on 2-cm-long roots and on the root hair cells of the induction zone. Supplementary Videos 1 and 2 show root hair cells displaying the NF-induced Ca^2+^ spiking in WT and spontaneous Ca^2+^ spiking in *cncg15a*^*GOF*^, respectively. Each video represents 9 min of pseudo-coloured cpVenus to display calcium concentration variation from low to high (green to red). Ca^2+^ oscillation traces were analysed as described previously^8^.

### Complementation of yeast lacking potassium transporters

DMI1 and DMI1^TVGYG^ were cloned via directional topoisomerase-based cloning (TOPO) cloning using primers P54/55 (Supplementary Table 1) into the pDONR207 vector according to Thermo Fisher Scientific manual’s instructions. They were subsequently subcloned into the yeast expression vector pAG414GAL-ccdB-HA (Addgene plasmid #14239) using LR Clonase II Enzyme mix (Invitrogen) according to the manufacturer’s instructions. The vectors were transformed into the MAB2d strain, which lacks the two potassium transporters Trk1 and Trk2 (*MATa ade2–1 can1–100 his3–11*,*15 leu2–3,112 trp1–1 ura3–1 mall0 ena1*Δ*::HIS3::ena4*Δ *nha1*Δ*::LEU2 trk1*Δ*::LEU2 trk2*Δ*::HIS3*) (ref. ^25^), via lithium acetate method^60^. The transformants were grown on a synthetic dropout (SD) medium lacking tryptophan (Trp) and supplied with 100 mM KCl, 2% glucose and 2% agar. The transformants were selected after 3 days of growth at 28 °C and subsequently inoculated in 5 ml liquid SD medium lacking Trp and supplied with 100 mM KCl, 1% sucrose and 1% galactose at 28 °C overnight. To perform the growth assay, the yeast cultures were centrifuged, washed in water and resuspended to an OD_600_ = 0.01 in 2.5 ml of SD 1% galactose and 1% raffinose medium lacking Trp and supplemented with the indicated concentration of KCl. The cultures were grown in a 12-well CytoOne plate (Starlab Ltd) at 28 °C in a shaker incubator (200 rpm) for 1 and 7 days, as indicated. The OD_600_ was measured using a BioPhotometer Model #6131 (Eppendorf).

### Wheat field trial

Field experiments were sown at the John Innes Centre experimental trial sites in Bawburgh, UK, at two different locations: 52° 37′ 50.7″ N, 1° 10′ 39.7″ E in 2023 and 52° 37′ 37.9″ N, 1° 10′ 54.0″ E in 2024. The soil of both fields is classified by the Land Information System at the Cranfield Environment Centre as slightly acidic loamy and clayey soils with impeded drainage. Each trial site was arranged in a split-plot design with two sections of 18 × 1.1 m in 2023, and 30 × 1.1 m in 2024. Commercial AM inoculum (Empathy rootgrow mycorrhizal fungi; Royal Horticultural Society) was applied to one section at a concentration of 1.11 kg m^−2^. A wide strip of 4 m separated the inoculated and non-inoculated sections to avoid contamination. In 2023, five independent replicates for each *Triticum* sp. WT/mutant plants were grown per section, and in 2024, three independent replicates for each *Triticum* sp. BC_3_ mutant NIL and WT NIL were grown per section. During the trial growing season, 2023 was slightly cooler than 2024, with lower average temperatures (2023, 11.6 °C; 2024, 12.1 °C). Both years experienced equivalent precipitation levels (2023, 156.4 mm; 2024, 165.9 mm), albeit 2023 had a 2-day event midway through the trial that accounted for 32% of the total rainfall. In terms of quantum radiation, 2023 had a higher average daily quantum radiation (435.8 μmol m^−2^ s^−1^) compared to 2024 (335.2 μmol m^−2^ s^−1^). Excel v.16.0 was used to calculate the average (Supplementary Table 3).

### Field soil analysis

To assess the soil nutrient quality of the field, 700 g of soil per sample was collected around the section at the same time as the section was dug out for AM analysis. The soil samples were analysed by Dove Associates Ltd. The amounts of Ca^2+^, sodium, aluminium, sulfur, potassium, magnesium, manganese, boron, copper, iron, zinc and molybdenum were assessed via inductively coupled plasma optical emission spectroscopy. Extractable phosphorus was measured via the Olsen extraction method. Available ammonium and nitrate were extracted with 2 M potassium chloride solution and measured by colorimetry (Supplementary Table 4). Excel v.16.0 was used to calculate the average.

### Histochemical GUS staining

*M. truncatula* roots were fixed in ice cold 90% methanol for 2 h at −20 °C. The roots were washed three times for 10 min in a reaction buffer containing 0.1 M Na_2_HPO_4_–NaH_2_PO_4_ (pH 7) and incubated in a reaction buffer supplemented with 1 mM K_3_Fe(CN)_6_, 1 mM K_4_Fe(CN)_6_, 5 mM NaEDTA, 0.1% Triton X-100 and 2 mM 5‐bromo‐4‐chloro‐3‐indolyl‐β‐d‐glucuronide. Samples were gently vacuum-infiltrated for 30 min and then incubated at 37 °C, in the dark, for 24 h. The samples were imaged with a DM6000 microscope (Leica) equipped with a DFC420 colour camera (Leica).

### Localization

*M. truncatula* A17 was transformed with *A.* *rhizogenes* AR1193 carrying the Golden Gate constructs *p35S:mCherry:T35S-pNOS:CNGC15c*^*GOF*^*:GFP:TNOS* or *p35S:mCherry:T35S*. Two-week-old transformed roots were analysed for green fluorescent protein (GFP) and mCherry fluorescence using the confocal laser scanning microscope Zeiss LSM 980 (GFP: excitation 488 nm and emission imaged between 500 and 530 nm; mCherry: excitation 587 nm and emission imaged between 600 and 620 nm).

### RNA-seq analysis

*M. truncatula* seedlings were grown on BNM agar medium supplemented with 0.1 µM l-α-(2-aminoethoxyvinyl) glycine for 24 h before treatment. The seedlings were incubated in six-well plates supplemented with liquid BNM with and without 10^−8^ M Nod factor, and with or without 3 mM KNO_3_^−^ for 3 h. Total RNA was extracted from root infection zones corresponding to 1 cm from the first elongated root hair upward (with RNeasy Plant Mini Kit; Qiagen) according to the manufacturer’s instructions. Genomic DNA was removed with TURBO DNA-free (Invitrogen) according to the manufacturer’s instructions. RNA library preparation and sequencing were performed by Novogene. RNA libraries were prepared with the Illumina TruSeq Stranded mRNA HT technology. RNA-seq was performed using the Illumina HiSeq platform with paired-end 150-bp (PE 150) strategy.

The resulting reads were quality controlled using FastQC v.0.11.8 and Trim Galore v.0.6.10, and mapped to *M.* *truncatula* v5 genome (MtrunA17r5.0-ANR) using STAR v.2.5.a. Gene read counts were retrieved using HTSeq v.0.9.1. Differentially expressed genes (DEGs) were identified by pairwise comparisons using DESeq2 v.3.18 package in R. DEGs with a false discovery rate-corrected *P* value < 0.05 were used for further analyses. Heat maps were generated in R using heat map package. Gene ontology over-representation analysis was performed with PANTHER v.18.0 online tool using the corresponding *M.* *truncatula* v4 gene IDs. Previously published RNA-seq reads used in this study were retrieved from the National Center for Biotechnology Information Sequence Read Archive (accession number SRP099836)^35^ and the Gene Expression Omnibus (accession number GSE154845)^34^.

### Gene expression analyses

RNA was extracted from *M.* *truncatula* root induction zone of 24-h-old seedlings after 3-h treatment, as indicated or root inoculated with *Sm*2011 for 21 days with RNeasy Plant Mini Kit (Qiagen) and subsequently treated with TURBO DNA-free (Invitrogen) before performing the reverse transcription with 1 µg RNA using SuperScript IV reverse transcriptase (Invitrogen). The quantitative gene expression was monitored with SYBR Green (Sigma-Aldrich) based quantitative PCR on a Bio-Rad thermocycler using gene specific primers. *UBC9* (*TC106312*)^61^ was used for normalization. The primers used for quantitative reverse transcription polymerase chain reaction (qRT-PCR) are listed in Supplementary Table 1.

### Phylogeny

CNGC15 sequences were identified through BLASTp and BLASTn v.2.13 searches against genomes on Phytozome v.13 (ref. ^62^) (Supplementary Table 5). A phylogenetic tree was constructed, as described in a previous study^8^. IQ-TREE (v.2.2.3) was used to construct the phylogenetic tree with the maximum likelihood approach. ModelFinder was used to find the best-fit evolutionary model (TPM2u+R5), and ultrafast bootstrap (UFBoot2) with 1,000 replications was used to estimate branch support^63^. Three figures were generated with the interactive Tree Of Life v.6 (ref. ^64^).

### Metabolite profiling analysis

Roots from *M.* *truncatula* inoculated with *S.* *meliloti* strain *Sm*2011 (OD_600_ = 0.01) for 21 days, *R.* *irregularis* for 5 weeks or non-inoculated were freeze dried and ground in liquid nitrogen. The root systems from two to three *M.* *truncatula* plants were used per biological replicate with a minimum of 36 mg ground root tissue used for analysis. Metabolite profiling was carried out by MS-Omics by Biogenity as follows. The analysis was carried out using a Vanquish LC (Thermo Fisher Scientific) coupled to a Orbitrap Exploris 240 mass spectrometer (Thermo Fisher Scientific). An electrospray ionization interface was used as ionization source. Analysis was performed in positive and negative ionization modes under polarity switching. The ultra-performance liquid chromatography–mass spectrometry (LC–MS) was performed, as described^65^ with the following modification. Peak areas were extracted using Compound Discoverer 3.3 (Thermo Fisher Scientific). Identification of compounds was performed at four levels. Level 1 is identification by retention times (compared against in-house authentic standards), accurate mass (with an accepted deviation of 3 ppm) and MS/MS spectra. Level 2a is identification by retention times (compared against in-house authentic standards) and accurate mass (with an accepted deviation of 3 ppm). Level 2b is identification by accurate mass (with an accepted deviation of 3 ppm) and MS/MS spectra. Level 3 is identification by accurate mass alone (with an accepted deviation of 3 ppm). All the raw data are presented in Supplementary Table 6.

#### Targeted metabolite analysis of wheat roots

The liquiritigenin analysis in 7-week-old wheat roots was carried out by Biogenity as follows. Methanol, formic acid, acetonitrile and the standard substances of liquiritigenin were obtained from Sigma-Aldrich. For the sample preparation, 0.1 g of the root samples was weighed, and 4 ml 80% MeOH was added. The samples were sonicated for 30 min at room temperature. The sonicated samples were centrifuged at 10,000 rpm for 10 min, and 1 ml of the clear supernatant was transferred to a new tube and dried using a vacuum concentrator. The dried extracts were reconstituted in 200 µl 80% MeOH and centrifuged at 10,000 rpm. The supernatant was transferred to a high-performance liquid chromatography (HPLC) vial for LC–MS analysis. LC–MS analysis was performed using a Vanquish Core HPLC system (Thermo Fisher Scientific) coupled with an Orbitrap Exploris 120 mass spectrometer (Thermo Fisher Scientific). A Waters ACQUITY UHPLC HSS T3 column (2.1 × 100 mm; 1.7 µm) was used for the separation. The mobile phases consisted of (A) water with 0.1% formic acid and (B) acetonitrile with 0.1% formic acid. The elution gradient was as follows: 0 min at 10% B, linearly increasing to 100% B over 10 min, maintaining 100% B for 2 min, returning to 10% B at 12.1 min and re-equilibrating at 10% B until 15 min. The flow rate was set at 0.3 ml min^−1^, and the column temperature was 40 °C. The injection volume was 2 µl. Orbitrap Exploris 120 was operated in the electrospray ionization (ESI) positive mode, spray voltage was 3.5 kV, sheath gas was 50 a.u., Aux gas 10 a.u., ion transfer tube temperature was set to 325 °C and vaporization temperature was set to 350 °C. For detection, a SIM scan was used with an isolation window of 2 *m*/*z*, resolution of 30,000 and centre mass of 257.0808 for liquiritigenin. For the data analysis and quantification, a stock solution of 1 mM was prepared for liquiritigenin in 80% MeOH. This solution was further diluted to obtain final concentrations of 1.58, 0.78, 0.39, 0.19, 0.097 and 0.048 µM to construct the calibration curve. The quantification was performed by linear regression of the peak area against concentration. Data processing and analysis were conducted using TraceFinder 5.1 (Thermo Fisher Scientific).

The retusin analysis in wheat roots was carried out as follows. *T.* *aestivum* cv. *Cadenza* WT and *Tacngc15a*^*GOF*^ were grown in 1 l 3:1 terragreen:sand in a controlled-environment greenhouse for 7 weeks. All harvested wheat roots were dried in VirTis freeze dryer, and then 200 mg dry weight root powder was mixed with 8 ml of 80% methanol (Sigma-Aldrich) containing 0.2 μM digitoxin (Sigma-Aldrich) as internal control and shaken at room temperature for 1 h. Samples were centrifuged at 13,000 rpm for 10 min. The supernatant was filtered through a Puradisc syringe filter, 0.45 µm, polytetrafluoroethylene (PTFE) membrane (Sigma-Aldrich). Samples were evaporated in Geneva HPLC mode overnight and redissolved in 200 μl methanol (Sigma-Aldrich). Samples were analysed on an ACQUITY Premier ultra-performance liquid chromatography equipped with a TQ Absolute tandem mass spectrometer (Waters). Chromatography was performed on a Kinetex EVO C18 column (100 × 2.1 mm, 2.6 μm, Phenomenex), remained 40 °C. The following gradient of 100% water with 0.2% formic acid (solvent A) versus acetonitrile with 0.2% formic acid (solvent B) was performed in the system: 0 min, 2% B; 10 min, 98% B; 11 min, 98% B, 11.1 min, 2% B; and 15 min, 2% B. The flow rate was 0.6 ml min^−1^, and the injection volume was 5 μl. MS detection was by positive mode electrospray monitoring the transition *m*/*z* 359 [M + H^+^] → 300.9 at a cone voltage of 52 and 32 V collision energy. The identity of retusin was confirmed by checking alternate fragments at *m*/*z* 106.8 (52 V collision energy), *m*/*z* 257.7 (44 V) and *m*/*z* 328.9 (28 V). Retention time was assessed for compound identification. The spray chamber conditions were 3 kV capillary voltage, 900 l h^−1^ drying gas at 500 °C and 150 l h^−1^ cone gas. To quantify retusin, 1 mM stock of retusin (Sigma-Aldrich) was dissolved in methanol. Serial dilutions (0–179.17 ng μl^−1^) of retusin were prepared in methanol containing 2 μM digitoxin. The quantification of the standard curve was analysed using MassLynx v.4.2 software.

### External flavonoid and flavonol applications

For *M.* *truncatula* nodulation assays, 1-week-old plants were transferred into terragreen/sand (Oil-Dri Ltd) to a ratio of 3:1 and watered with either water or water containing 0.1 µM naringenin, 0.1 µM liquiritigenin or 0.1 µM retusin. After 2 days, all plants were inoculated with *Sm*2011 (OD_600_ = 0.01). The plants were grown in a controlled-environment room at 22 °C (80% humidity, 16-h photoperiod and 300 µmol m^−2^ s^−1^) and were watered twice a week with water with or without 0.1 µM naringenin (Sigma-Aldrich), 0.1 µM liquiritigenin (Sigma-Aldrich) or 0.1 µM retusin (Sigma-Aldrich). Nodules were scored for small and mature nodules 14 days post-inoculation with *Sm*2011.

For *M.* *truncatula* mycorrhization experiments, 7-day-old seedlings were planted into 84% 1:1 sand/terragreen (Oil-Dri Ltd) and 16% mycorrhizal inoculum (homogenized soil substrate containing *A.* *schoenoprasum* roots colonized by *R.* *irregularis* DAOM197198) and watered with either water or water containing 0.1 µM naringenin, 0.1 µM liquiritigenin or 0.1 µM retusin. Plants were grown in a controlled-environment room at 22 °C (80% humidity, 16-h photoperiod and 300 µmol m^−2^ s^−1^) and were watered twice a week with addition water or 0.1 µM naringenin, 0.1 µM liquiritigenin or 0.1 µM retusin solution.

For *Triticum* sp. mycorrhization experiments, 5-day-old seedlings were transferred in 80% 1:1 sand:terragreen mix and 20% mycorrhizal inoculum. Wheat plants were grown in a controlled-environment room at 22 °C (35% daytime humidity and 50% night-time humidity, 16-h photoperiod and 500 μmol m^−2^ s^−1^) and watered with water with or without 0.1 µM retusin twice a week.

### Statistical analyses

Statistical significance was determined by two-tailed unpaired *t*-test with a previous *F*-test for homoscedasticity, one-way ANOVA followed by post hoc, as indicated using GraphPad Prism version 8 unless stated otherwise. Dot plots were used to show individual points. *P* values over 0.05 were considered nonsignificant. For metabolite analysis, the data were filtered, so only metabolites with at least 60% valid values across all the samples or 70% within an experimental group were used in the downstream data analysis. Filtration and statistical analysis were performed in the R programming by Biogenity using limma v.3.18 (ref. ^66^).

### Accession numbers

Accession of the sequences from this study are listed in Supplementary Tables 2 and 5.

### Reporting summary

Further information on research design is available in the Nature Portfolio Reporting Summary linked to this article.

## Online content

Any methods, additional references, Nature Portfolio reporting summaries, source data, extended data, supplementary information, acknowledgements, peer review information; details of author contributions and competing interests; and statements of data and code availability are available at 10.1038/s41586-024-08424-7.

## Supplementary information

Supplementary Figures Supplementary Figs. 1–3.

Reporting Summary

Supplementary Table 1 Primers used in this study.

Supplementary Table 2 Amino acid sequences used to assess S1 conservation.

Supplementary Table 3 Daily average air temperature (°C), precipitation (mm) and quantum radiation (μmol m^−2^ s^−1^) from 2023 and 2024 experimental field sites.

Supplementary Table 4 Soil analysis of the field trial 2023.

Supplementary Table 5 Amino acid sequences used for phylogenetic analysis of wheat CNGC15.

Supplementary Table 6 Raw data of metabolite profiling.

Supplementary Video 1 NF-induced Ca^2+^ spiking in WT root hair cell.

Supplementary Video 2 Spontaneous Ca^2+^ spiking in *cncg15a*^*GoF*^ root hair cell.

## Source data

Source Data Fig. 1

Source Data Fig. 2

Source Data Fig. 3

Source Data Fig. 4

Source Data Extended Data Fig. 1

Source Data Extended Data Fig. 3

Source Data Extended Data Fig. 4

Source Data Extended Data Fig. 5

Source Data Extended Data Fig. 8

Source Data Extended Data Fig. 9

Source Data Extended Data Fig. 10

## Data Availability

Raw metabolite data are presented in Supplementary Table 6. Raw RNA-seq reads are available at the European Nucleotide Archive (accession number PRJEB80093). Previously published RNA-seq reads used in this study from 10.1186/s12915-022-01450-9 and 10.1126/science.aan0081 are available at https://www.ncbi.nlm.nih.gov/geo/query/acc.cgi?acc=GSE154845 and https://www.ncbi.nlm.nih.gov/sra/?term=SRP099836, respectively. Accession numbers of all sequences as well as the database used to retrieve them are listed in Supplementary Tables 2 and 5. *M.* *truncatula* sequence IDs are from Mt4.0v1, and wheat sequences are from the International Wheat Genome Sequencing Consortium RefSeq v.1.1. Seed materials are available at https://www.jic.ac.uk/research-impact/technology-research-platforms/molecular-genetics/medicago-truncatula/ and https://www.seedstor.ac.uk/search-browseaccessions.php?idCollection=24. Source data are provided with this paper.

## Code availability

The code used for the RNA-seq analysis is available on GitHub at https://github.com/charpenm/RNAseq-analysis.
