# Supplementary material for: Autoactive CNGC15 enhances root endosymbiosis in legume and wheat
Source: Nature. 2025 Jan 15;638(8051):752–9. doi: 10.1038/s41586-024-08424-7 (PMC11839481; doi:10.1038/s41586-024-08424-7)
Supplement: Supplementary file 1 — Supplementary Figs. 1–3. [file 41586_2024_8424_MOESM1_ESM.pdf]

nature portfolio

https://doi.org/10.1038/s41586-024-08424-7

Supplementary information

Autoactive CNGC15 enhances root endosymbiosis in legume and wheat

In the format provided by the authors and unedited

Transmembrane domain S1

```
MtCNGC15a       PRINLWNKIFLAACLISLFVDPLFFYL     103
MtCNGC15c       QLIHRWNKIFLVACLVSLFVDPLFFYL     109
MtCNGC15b       QTIHQWNKIFLVACLISLFVDPLFFYL     91
GmCNGC15a       HRVNTWNKIFLAACLLSLFVDPLFFYL     103
GmCNGC15b       HRVNTWNKIFLAACLLSLFVDPLFFYL     107
GmCNGC15d       QTIHRWNKIFLVACLVSLFVDPLFFYL     109
GmCNGC15e       QTIHRWNKIFLVACLASLFVDPLFFYL     109
GmCNGC15c       QTIHRWNKILLVACLVSLFVDPLFFYL     106
SlCNGC15a       RLINIWNKCFLIASLTSLFVDPLFFYL     109
SlCNGC15b       HVIRRWNKFFLVSCLVSLFVDPLLFYL     111
SlCNGC15c       PTVRRWNKILLVACLIGLFVDPLFFYL     111
TaCNGC15a-A1    QVVHRWHKVFLAACLASLFVDPLFLYL     116
TaCNGC15a-B1    QVIHRWHKVFLAACLASLFVDPLFLYL     116
TaCNGC15a-D1    QVIHRWHKVFLAACLASLFVDPLFLYL     116
TaCNGC15b-A1    RVIHLWNKIFLTACLLSLFVDPLFLYL     127
TaCNGC15b-B1    RVIHLWNKIFLTACLLSLFVDPLFLYL     126
TaCNGC15b-D1    RVIHLWNKIFLTACLLSLFVDPLFLYL     128
OsCNGC15        QLIHLWSKIFLAACLASLFVDPLFLYL     120
ZmCNGC15        QLIHLWNKIFLSACLLSLFVDPLFLYL     119
A.hCNGC15a      PRVNRWNRIFLVACLISLFVDPLFFYL     98
A.hCNGC15c      QAIHRWNKIFLVASLVSLFVDPLFFYL     67
A.hCNGC15b      QTIHRWNKIFLVACLVSLFVDPLFFYL     88
AtCNGC15        QTIRRWNKIFLIACLVSLFVDPLFFFL     100
AtCNGC2         KRVQRWNRALLLARGMALAVDPLFFYA     145
MtCNGC2         KRVQLSNRALLLARGVALAIDPLFFYA     141
AtCNGC4         KWVREWNKVFLLVCATGLFVDPLFLYT     111
MtCNGC4a        KWAEEWNRVFLLVCAMGLFVDPLFFYA     95
MtCNGC4b        KWVQEWNRVFLLVCAAGLFVDPLFFYA     94
MtCNGCIVAe      KVVHLWNKFLAIICFVAIFVDPLFLFS     36
AtCNGC19        KFVQVWTRVLAFSSLVAIFIDPLFFFL     120
AtCNGC20        KEVQTWTKFFALSCLLAIFIDPLFFFL     112
MtCNGCIVAa      KLVHQWNKFFAICCLVAIFVDPLFFFL     124
MtCNGCIVAd      KVVQQWNTIFAISCSVAIFFDPLFFFL     59
MtCNGCIVAf      KLVQQWNKFLAIFCMLAIFVDPLFLFL     65
MtCNGCIVAb      KIVQQWNKFLAIFCLLAIFVDPLFFFL     100
MtCNGCIVAc      KVVQQWNKFLAIFCIVAIYVDPLFFFL     109
AtCNGC14        DAVLQWNRVFLFWCLVALYVDPLFFFL     104
AtCNGC17        EIVLKWNWVFIVSCMVALFIDPLYFFV     103
MtCNGC14        DIFLEWKRAFLCSCILSLFVDPLFFYL     105
MtCNGC17        QIVLKWNRLFIVSCLLALFVDPLYFYL     75
AtCNGC16        DLITRWNHIFLITCLLALFLDPLYFYL     76
MtCNGC16        PFVAKWNVTFLYACLFALFLDPLYFYI     67
AtCNGC18        NIVTYWNHVFLITSILALFLDPFYFYV     71
MtCNGC18        ELVAYWNKVFLVTSLLALFIDPLYFFL     75
AtCNGC7         KTLLVWNRLFVISCILAVSVDPLFFYL     93
AtCNGC8         KTLLLWNRMFVISCILAVSVDPLFFYL     129
AtCNGC6         KFLLLCNKLFVASCILAVSVDPLFLYL     135
AtCNGC9         KFLLLCNKLFVTSCILAVSVDPLFLYL     109
AtCNGC5         KFLLYCNKLFVASCILSVFVDPFFFYL     120
MtCNGCII        KFLLLWNKLFVISCIFSVFVDPLFFYL     131
AtCNGC10        SFLQNWNKIFLFACVVALAIDPLFFYI     100
AtCNGC13        SFLQNWNKIFLFASVIALAIDPLFFYI     100
AtCNGC3         SYLQSWNKIFLLLSVVALAFDPLFFYI     104
AtCNGC11        KTLENWRKTVLLACVVALAIDPLFLFI     61
AtCNGC12        KTLENWRKTVLLACVVALAIDPLFLFI     61
AtCNGC1         PFLQRWNKIFVLACIIAVSLDPLFFYV     116
MtCNGC1a        TFLQKWNKIFVLLCVIAVSLDPLFFYV     115
MtCNGC1b        PFLQKWNKIFVLSCLIAVSIDPLFFYI     113
MtCNGCIa        PLLQKWNKIFVITCVMAVSMDPLFFYI     93
MtCNGCIb        PMLQKWNKIFVITCVLAISVDPFFFYI     107
                     .      .: .**: ::
```

SI Fig. 1| Sequence conservation of S1 amino acid sequence of CNGC15.
Protein sequence alignment of the S1 transmembrane domain of CNGC15 from *Medicago truncatula (*Mt*)*, *Arabidospis thaliana* (At), *Arachis hypogaea* (A.h), *Glycine max* (Gm), *Oryza sativa* (Os), *Solanum lycopersicum* (Sl), *Triticum aesitivum* (Ta) and *Zea mays* (Zm)*.* Highly conserved Proline residue mutated in CNGC15^{GOF} is highlighted in yellow.

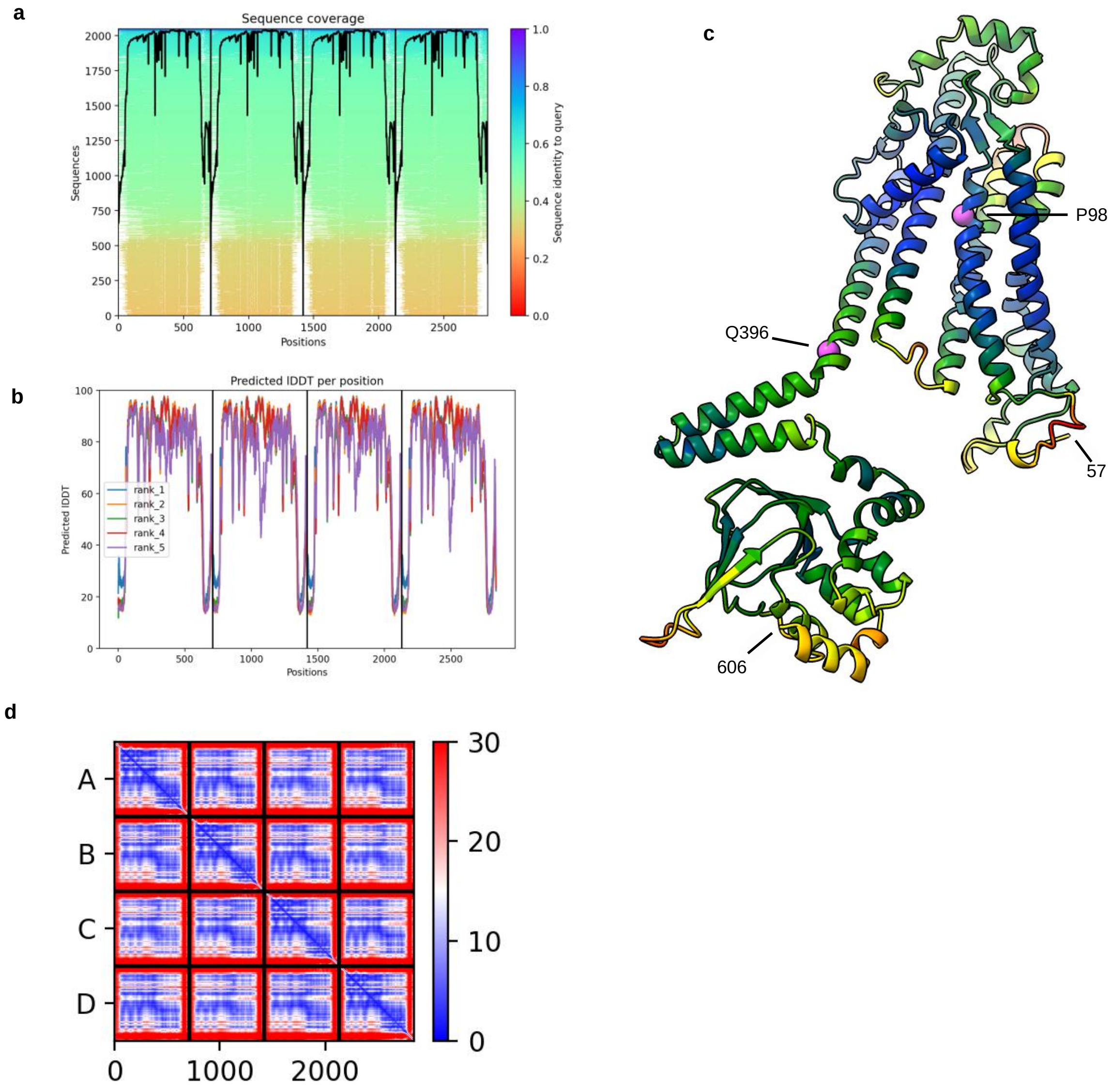

SI Fig. 2| Confidence metrics of CNGC15a homotetramer.

a, Sequence identity plot showing good coverage and depth for the multiple sequence alignment against the MtCNGC15a sequence (the alignment is shown four times, once for each subunit). **b,** Plots of the predicted local distance difference test score (pLDDT) for the five independent tetramer models, which are overlaid (see inset colour scheme). The scores for each subunit are shown in separate panels. pLDDT values exceeding 70 are achieved across all the five models, except at the termini and in a few surface loops. **c**, Alphafold2 model of a single subunit taken from the top ranked model coloured according to pLDDT score, where residues with values of less than 50 are coloured red; residues with values in the range 50-100 are shown in rainbow colouration from red through to blue, with blue indicating the highest quality. Residues at the poorly predicted termini were omitted (i.e. residues 1-56 and 607-710). Magenta spheres indicate the position of Pro98 positioned in S1 and the channel gating residues Gln396. **d**, Visualization of Predicted Aligned Errors (PAE) for the top ranked model; the off-diagonal blue blocks indicate high confidence in the relative placement of the subunits within the tetramer. A to D indicate the four units of MtCNGC15. The x-axis numbering in **a**, **b** and **d** indicates the cumulative residue count for the tetramer (total = 4 x 710 = 2840 residues).

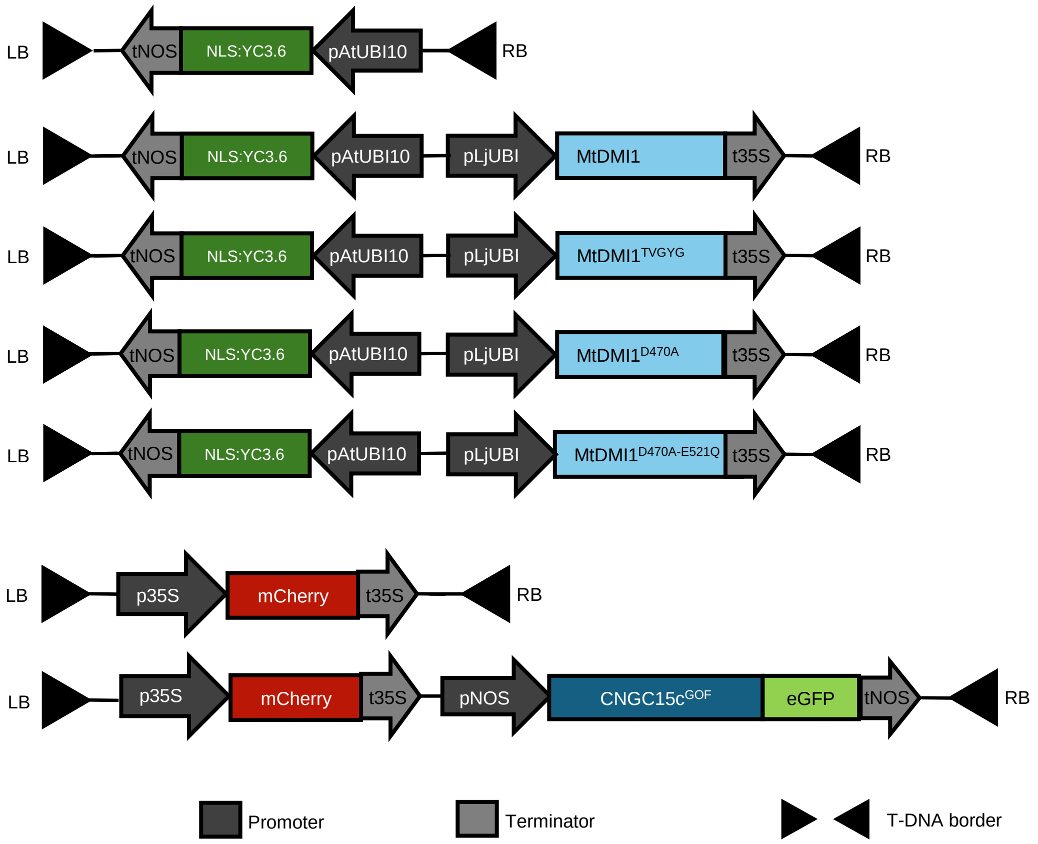

SI Fig. 3| GoldenGate constructs generated in this study.
