# Supplementary material for: Autoactive CNGC15 enhances root endosymbiosis in legume and wheat
Source: Nature. 2025 Jan 15;638(8051):752–9. doi: 10.1038/s41586-024-08424-7 (PMC11839481; doi:10.1038/s41586-024-08424-7)
Supplement: Supplementary file 2 — Reporting Summary [file 41586_2024_8424_MOESM2_ESM.pdf]

nature portfolio

Corresponding author(s): Myriam Charpentier

Last updated by author(s): Oct 29, 2024

Reporting Summary

Nature Portfolio wishes to improve the reproducibility of the work that we publish. This form provides structure for consistency and transparency in reporting. For further information on Nature Portfolio policies, see our Editorial Policies and the Editorial Policy Checklist.

Statistics

For all statistical analyses, confirm that the following items are present in the figure legend, table legend, main text, or Methods section.

n/a	Confirmed	
☐	☒	The exact sample size (*n*) for each experimental group/condition, given as a discrete number and unit of measurement
☐	☒	A statement on whether measurements were taken from distinct samples or whether the same sample was measured repeatedly
☐	☒	The statistical test(s) used AND whether they are one- or two-sided<br>*Only common tests should be described solely by name; describe more complex techniques in the Methods section.*
☒	☐	A description of all covariates tested
☐	☒	A description of any assumptions or corrections, such as tests of normality and adjustment for multiple comparisons
☐	☒	A full description of the statistical parameters including central tendency (e.g. means) or other basic estimates (e.g. regression coefficient) AND variation (e.g. standard deviation) or associated estimates of uncertainty (e.g. confidence intervals)
☐	☒	For null hypothesis testing, the test statistic (e.g. *F*, *t*, *r*) with confidence intervals, effect sizes, degrees of freedom and *P* value noted<br>*Give P values as exact values whenever suitable.*
☒	☐	For Bayesian analysis, information on the choice of priors and Markov chain Monte Carlo settings
☒	☐	For hierarchical and complex designs, identification of the appropriate level for tests and full reporting of outcomes
☒	☐	Estimates of effect sizes (e.g. Cohen's *d*, Pearson's *r*), indicating how they were calculated

Our web collection on statistics for biologists contains articles on many of the points above.

Software and code

Policy information about availability of computer code

Data collection	Illumina HiSeq (PE150), Vanquish LC-Orbitrap Exploris 240MS, capillary ABI3730 sequencer, Vanquish Core HPLC system-Orbitrap Exploris 120MS, Acquity Premier UPLC-TQ-Absolute tandem MS
Data analysis	MUSCLE v3.8.425, ColabFold (version 1.5.2), COOT software (version 1), FastQC (version 0.11.8), TrimGalore (version 0.6.10), STAR-2.5a, PANTHER (version 18.0), DEseq2 (version 3.18), BLAST (version 2.13), Phytozome (version13), IQ-Tree (version 2.2.3), Tree Of Life (version 6), limma (version 3.18), GraphPad Prism (version 8), Metafluor (version 7.0), Excel (version 16.0), ChimeraX v1.5, htseq 0.9.1, Compound Discoverer v3.3, TraceFinder v5.1, MassLynx v4.2, https://github.com/charpenm/RNAseq-analysis.

For manuscripts utilizing custom algorithms or software that are central to the research but not yet described in published literature, software must be made available to editors and reviewers. We strongly encourage code deposition in a community repository (e.g. GitHub). See the Nature Portfolio guidelines for submitting code & software for further information.

Data

Policy information about availability of data

All manuscripts must include a data availability statement. This statement should provide the following information, where applicable:

- Accession codes, unique identifiers, or web links for publicly available datasets
- A description of any restrictions on data availability
- For clinical datasets or third party data, please ensure that the statement adheres to our policy

All data generated during this study are provided in the source data files. Raw metabolite data are presented in SI Table 6. Raw RNAseq reads are available at European Nucleotide Archive (accession number PRJEB80093). Previously published RNAseq reads used in this study from doi.org/10.1186/s12915-022-01450-9 and DOI:10.1126/science.aan0081 are available at https://www.ncbi.nlm.nih.gov/geo/query/acc.cgi?acc=GSE154845, and https://www.ncbi.nlm.nih.gov/sra/?term=SRP099836, respectively. Accession of all sequences as well as database used to retrieve them of are listed in SI Table 2 and 5. M. truncatula sequences ID are from Mt4.0v1, and wheat sequences from IWGSC RefSeq v1.1. Seeds material are available at http://revgenuk.jic.ac.uk/order.htm and https://www.seedstor.ac.uk/search-browseaccessions.php?idCollection=24.

Research involving human participants, their data, or biological material

Policy information about studies with human participants or human data. See also policy information about sex, gender (identity/presentation), and sexual orientation and race, ethnicity and racism.

Reporting on sex and gender	not applicable
Reporting on race, ethnicity, or other socially relevant groupings	not applicable
Population characteristics	not applicable
Recruitment	not applicable
Ethics oversight	not applicable

Note that full information on the approval of the study protocol must also be provided in the manuscript.

Field-specific reporting

Please select the one below that is the best fit for your research. If you are not sure, read the appropriate sections before making your selection.

☒ Life sciences ☐ Behavioural & social sciences ☐ Ecological, evolutionary & environmental sciences

For a reference copy of the document with all sections, see nature.com/documents/nr-reporting-summary-flat.pdf

Life sciences study design

All studies must disclose on these points even when the disclosure is negative.

Sample size	No calculation was performed to pre-determine sample size. The sample size was chosen as large as possible and in line with the field and determined by previous experience in performing experiments of the same technical nature. Given the sample size, adequate statistical analysis was performed.
Data exclusions	No data were excluded.
Replication	Independent biological replicate were performed in all experiments, with all replications successful. For most of the experiments, the biological replicates were performed by 2 to 3 independent people.<br>The field trials were performed over two growing season in two different locations and in 5 different plots per location in 2023, and 3 different plots in 2024, with all replications successful.
Randomization	For each wheat field trial, the different plots were randomly distributed across the field, and plants were randomly selected for phenotyping within these plots. Calcium imaging on different genotypes is performed randomly. With the exception of the blinding experiments of Extended Data fig 1c and Extended data figure 3d, all materials were known to be homozygous. Therefore, individual organisms were randomly assigned to control and treatment groups.
Blinding	Blinding was applied in the nodulation phenotyping of Extended Data Fig. 1c,d and calcium analysis of the extended Data Fig. 3d. All other phenotyping experiments were not blinded, but number were assigned to each tube and reconcile with samples names once phenotyping was completed.

Reporting for specific materials, systems and methods

We require information from authors about some types of materials, experimental systems and methods used in many studies. Here, indicate whether each material, system or method listed is relevant to your study. If you are not sure if a list item applies to your research, read the appropriate section before selecting a response.

Materials & experimental systems

n/a	Involved in the study
☒	☐ Antibodies
☒	☐ Eukaryotic cell lines
☒	☐ Palaeontology and archaeology
☒	☐ Animals and other organisms
☒	☐ Clinical data
☒	☐ Dual use research of concern
☐	☒ Plants

Methods

n/a	Involved in the study
☒	☐ ChIP-seq
☒	☐ Flow cytometry
☒	☐ MRI-based neuroimaging

Dual use research of concern

Policy information about dual use research of concern

Hazards

Could the accidental, deliberate or reckless misuse of agents or technologies generated in the work, or the application of information presented in the manuscript, pose a threat to:

No	Yes
☒	☐ Public health
☒	☐ National security
☒	☐ Crops and/or livestock
☒	☐ Ecosystems
☒	☐ Any other significant area

Experiments of concern

Does the work involve any of these experiments of concern:

No	Yes
☒	☐ Demonstrate how to render a vaccine ineffective
☒	☐ Confer resistance to therapeutically useful antibiotics or antiviral agents
☒	☐ Enhance the virulence of a pathogen or render a nonpathogen virulent
☒	☐ Increase transmissibility of a pathogen
☒	☐ Alter the host range of a pathogen
☒	☐ Enable evasion of diagnostic/detection modalities
☒	☐ Enable the weaponization of a biological agent or toxin
☒	☐ Any other potentially harmful combination of experiments and agents

Plants

Seed stocks	Seeds are available at http://revgenuk.jic.ac.uk/order.htm or https://www.seedstor.ac.uk/shopping-cart-tilling.php
Novel plant genotypes	All gain of function mutants were identified by reverse screening the available TILLING plant collections at RevGenUK and http://www.wheat-tilling.com/
Authentication	Segregating population analysis was conducted to assess the phenotype of the gain-of-function mutant, as well as an analysis of the same mutation in a different CNGC15 paralogue, and species and plant cultivar.
